# Ribosome stalling during *c-myc* translation presents actionable cancer cell vulnerability

**DOI:** 10.1093/pnasnexus/pgae321

**Published:** 2024-08-13

**Authors:** Tejinder Pal Khaket, Suman Rimal, Xingjun Wang, Sunil Bhurtel, Yen-Chi Wu, Bingwei Lu

**Affiliations:** Department of Pathology and Programs in Neuroscience and Cancer Biology, Stanford University School of Medicine, Stanford, CA 94305, USA; Department of Pathology and Programs in Neuroscience and Cancer Biology, Stanford University School of Medicine, Stanford, CA 94305, USA; Department of Pathology and Programs in Neuroscience and Cancer Biology, Stanford University School of Medicine, Stanford, CA 94305, USA; Department of Pathology and Programs in Neuroscience and Cancer Biology, Stanford University School of Medicine, Stanford, CA 94305, USA; Department of Pathology and Programs in Neuroscience and Cancer Biology, Stanford University School of Medicine, Stanford, CA 94305, USA; Department of Pathology and Programs in Neuroscience and Cancer Biology, Stanford University School of Medicine, Stanford, CA 94305, USA

**Keywords:** cMyc, cancer, translation stalling, ribosome-associated quality control (RQC), Nsp1

## Abstract

Myc is a major driver of tumor initiation, progression, and maintenance. Up-regulation of Myc protein level rather than acquisition of neomorphic properties appears to underlie most Myc-driven cancers. Cellular mechanisms governing Myc expression remain incompletely defined. In this study, we show that ribosome-associated quality control (RQC) plays a critical role in maintaining Myc protein level. Ribosomes stall during the synthesis of the N-terminal portion of cMyc, generating aberrant cMyc species and necessitating deployment of the early RQC factor ZNF598 to handle translational stress and restore *cMyc* translation. ZNF598 expression is up-regulated in human glioblastoma (GBM), and its expression positively correlates with that of cMyc. ZNF598 knockdown inhibits human GBM neurosphere formation in cell culture and Myc-dependent tumor growth in vivo in *Drosophila*. Intriguingly, the SARS-COV-2-encoded translational regulator Nsp1 impinges on ZNF598 to restrain cMyc translation and consequently cMyc-dependent cancer growth. Remarkably, Nsp1 exhibits synthetic toxicity with the translation and RQC-related factor ATP-binding cassette subfamily E member 1, which, despite its normally positive correlation with cMyc in cancer cells, is co-opted by Nsp1 to down-regulate cMyc and inhibit tumor growth. Ribosome stalling during *c-myc* translation thus offers actionable cancer cell vulnerability.

**Significance Statement**

Myc is a major driver of tumor initiation, progression, and maintenance. Up-regulation of Myc level appears to underlie most Myc-driven cancers. Cellular mechanisms governing Myc expression remain incompletely understood. In this study, we show that ribosome-associated quality control (RQC) critically maintains cMyc protein level and is particularly required in cancer cells. Ribosomes stall during the synthesis of the N-terminus of cMyc, generating aberrant cMyc species and necessitating deployment of the RQC machinery to restore *cMyc* translation and handle translational stress. Genetic manipulation of ZNF598 and other components of the RQC pathway inhibits human glioblastoma neurosphere formation in culture and Myc-dependent tumor growth in vivo in animal models. Ribosome stalling during *c-myc* translation thus offers an actionable cancer vulnerability.

## Introduction

Myc is a master regulator of gene expression in normal cells and is dysregulated in ∼70% of human cancers (1, 2). Myc drives tumor initiation, progression, and maintenance, making it an appealing therapeutic target (3–5). However, conventional drug targeting of Myc has been challenging due to its intrinsically disordered structure and the absence of druggable pockets (6, 7). As the up-regulation of Myc expression rather than acquisition of neomorphic properties underlies most Myc-driven cancer (1, 4), it is imperative to understand how high Myc expression is achieved in disease state.

The translation of mRNAs is tightly regulated and constantly surveyed for errors. During translation elongation, ribosome slowdown and stalling can occur for various reasons. Some are functional, whereas others are detrimental and can be triggered by damaged mRNAs, mRNA secondary structures, insufficient supply of aminoacyl-tRNAs, or environmental stress (8, 9). Ribosome slowdown and stalling can result in ribosome collision (10), which is sensed by cells as a proxy for aberrant translation and can trigger quality control process associated with stalled ribosome (11–15). Key factors involved in this ribosome-associated quality control (RQC) process include the ubiquitin ligase ZNF598 and the 40S subunit protein Rack1, which recognize the distinct 40S–40S interface of collided ribosomes and promote ubiquitination of specific 40S proteins (16, 17), and the ASC complex, which disassembles the leading collided ribosome (18, 19). This triggers a series of downstream events, including ribosome subunit splitting and recycling by ATP-binding cassette subfamily E member 1 (ABCE1) (20), C-terminal Ala and Thr addition (CAT-tailing) of nascent peptide chains (NPCs) stalled on 60S subunit (21, 22), and degradation of stalled NPC and mRNA. The importance of this ribosome-mediated QC process is highlighted by the findings that RQC factors regulating translation elongation and termination are critical for neuronal function and integrity (23–25) and that inefficient RQC results in translation stalling and ensuing accumulation of faulty translation products that perturb proteostasis and contribute to the pathogenesis of Alzheimer's disease (AD), Parkinson's disease, and amyotrophic lateral sclerosis (26–28).

Whether the RQC pathway is critically involved in cancer biology remains underexplored. In this study, we show that RQC plays an important role in regulating Myc expression in cancer cells as well as noncancer cells. We found that ribosomes stall during the synthesis of the N-terminal portion of cMyc, necessitating the deployment of the RQC pathway to restore *cMyc* translation and handle translational stress. Genetic manipulation of the early RQC factor ZNF598 and other RQC factors strongly influences human glioblastoma (GBM) neurosphere formation in vitro and Myc-dependent tumor growth in vivo in *Drosophila* and mouse models, indicating particular dependence of cancer cells on the quality control of stalled *cMyc* translation. Together, our results reveal that ribosome stalling during *c-myc* translation presents cancer cell vulnerability that may be targeted for therapeutic purpose.

## Results

### SARS-CoV-2-encoded translational regulator Nsp1 down-regulates cMyc protein level

**Fig. 1.**
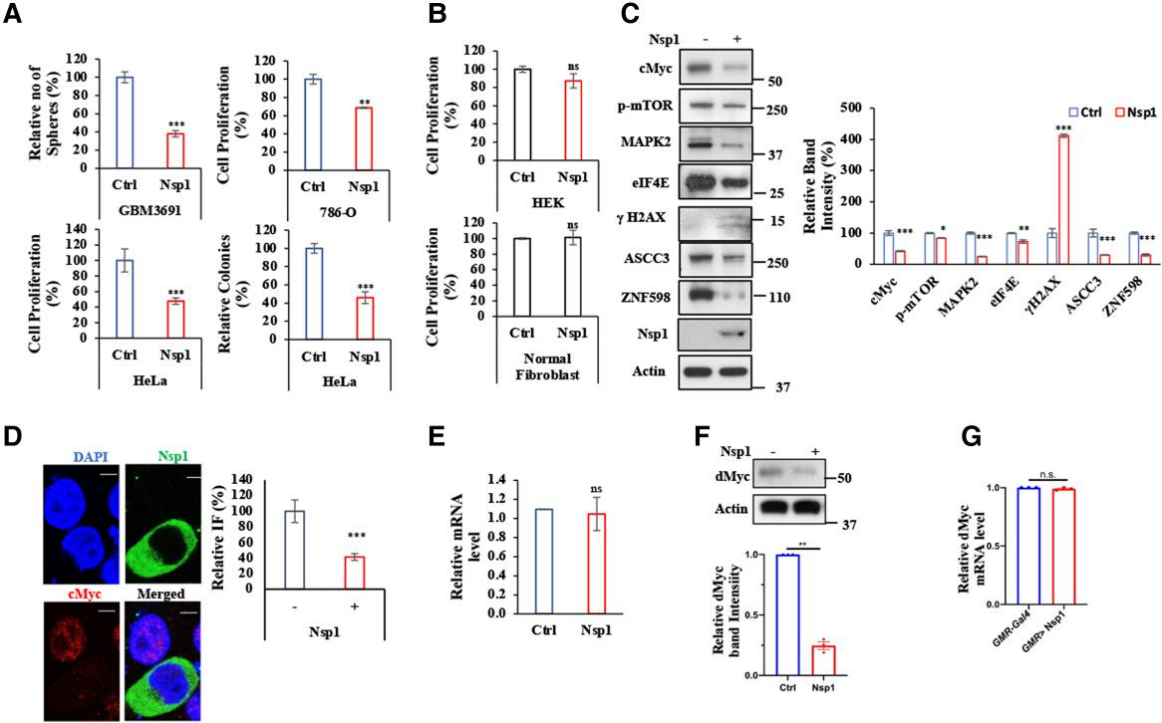
Inhibition of cMyc by Nsp1 in cancer cells. A) Effect of Nsp1 expression on cancer cell GBM 3691, HeLa, and 786-O proliferation was measured by sphere formation or MTT assays. Colony formation assay was also performed in Nsp1-transfected HeLa cells. B) Normal cells are relative resistant to Nsp1-induced growth inhibition. Similar amount of Nsp1 plasmid that caused toxicity in cancer cells as shown in (A) was transfected into normal fibroblasts and HEK293 cells and cell proliferation was measured by MTT assay. C) Immunoblots and data quantification of cMyc, p-mTOR, MAPK2, eIF4E, p-H2AX, ASCC3, and ZNF598 levels in Nsp1 expressing HeLa cells. D) Immunofluorescent staining and quantification of cMyc level in NSP1-expressed HeLa cells. cMyc was labeled with Alexa Fluor 633 fluorescence and counterstained with Alexa Fluor 488 for Nsp1 and DAPI (scale bar = 5 µm). E) qRT-PCR of *c-myc* mRNA level in Nsp1 expressing cells. F) Immunoblots for dMyc in *GMR* > *InR* and *GMR* > *InR/Nsp1* fly heads. G) Effect of Nsp1 expression on *dMyc* mRNA level in *GMR* > *InR* and *GMR* > *InR/Nsp1* fly heads. Actin serves as loading control in immunoblots. The results were quantified and statistically analyzed by GraphPad Prism software (****P < 0.0001, ***P < 0.001, **P < 0.01, *P < 0.05, n.s., not significant, in Student's t tests or one-way ANOVA test followed by Student–Newman–Keuls posttest).

SARS-CoV-2-encoded Nsp1 suppresses host gene expression by ribosome association and inhibition of mRNA translation (29, 30). It remains unclear whether Nsp1 acts as a general repressor of mRNA translation (31, 32), as there is evidence suggesting that Nsp1 may positively regulate the translation of SARS-CoV-2 viral mRNA (33) and some host protein levels may be positively regulated by Nsp1 (34). In mammalian cell culture studies of Nsp1, we noticed that it led to cytotoxicity in several cancer cell lines, including GBM cells (Fig. 1A). To test whether this effect was specific to cancer cells, we expressed Nsp1 in noncancer cell lines. Nsp1 exhibited low or no toxicity in HEK293 cells and human fibroblasts cells when similar amount of plasmid that caused toxicity in cancer cells was introduced (Fig. 1B). In dose–response studies, cytotoxicity as measured by the MTT (3-(4,5-dimethylthiazol-2-yl)-2,5-diphenyltetrazolium bromide) assay was observed in noncancer cells only when excess plasmid was transfected (Fig. S1A). HEK293 cells were sensitive to Nsp1 only when higher amounts of plasmids were transfected, whereas normal human fibroblasts were insensitive at all Nsp1 levels tested. Nsp1 expression in cancer cells resulted in the down-regulation of several oncogenic or progrowth signaling pathways, including the mTOR and MAPK pathways, and up-regulation of the DNA damage marker p-H2AX (Figs. 1C and S1B), consistent with its anticancer activity. In particular, we found that the protein levels of cMyc and cMyc targets such as eIF4E, as well as exogenously expressed cMyc, were significantly reduced by Nsp1 (Figs. 1C and S1B, C). cMyc reduction by Nsp1 was confirmed by immunostaining of Nsp1-transfected cells when compared with nontransfected cells (Fig. 1D). The Nsp1-induced down-regulation of cMyc protein level was not correlated with mRNA level change (Fig. 1E), suggesting that Nsp1 regulates cMyc at the translational or posttranslational level. Consistent with this notion, an Nsp1 mutant containing the K164A/H165A (Nsp1-KH) mutations that are defective in ribosome binding and translational regulation (34) failed to reduce cMyc level (Fig. S1D).

We previously showed that Nsp1 ameliorates the toxicity caused by stalled translation of amyloid precursor protein (APP) or the C-terminal fragment of APP (APP.C99) linked to AD (34). In an RNA-seq analysis aimed to assess gene expression changes induced by Nsp1 in APP.C99 expressing flies, we noticed that genes involved in translation, ribosome biogenesis, mitochondrial quality control and fission/fusion, mitochondrial biogenesis (e.g. respiratory chain assembly, CoQ biosynthesis, and TOM/TIM import machinery), and transmembrane transport were down-regulated, whereas those involved in innate immunity, Dorsal/Toll signaling, stress response, ubiquitin proteosome system/proteolysis, chemo/gustatory response, and differentiation were up-regulated (Fig. S1E). Intriguingly, many of these Nsp1 regulated genes were previously shown to be inversely regulated by cMyc (35, 36). This finding in APP.C99 expressing flies, though not in a cancer context, provided further support for Nsp1 antagonism of Myc function in an in vivo setting and may be relevant to the regulation of cMyc protein level in normal as well as cancer cells. Consistently, as we observed in cancer cell lines, endogenous dMyc level was reduced by Nsp1 in flies (Fig. 1F), and this occurred in the absence of obvious *dMyc* mRNA level change (Fig. 1G). These results suggest that Myc expression can be regulated at the translational or posttranslational level in normal tissues and cancer cells.

### Ribosome stalling during *cMyc* translation

**Fig. 2.**
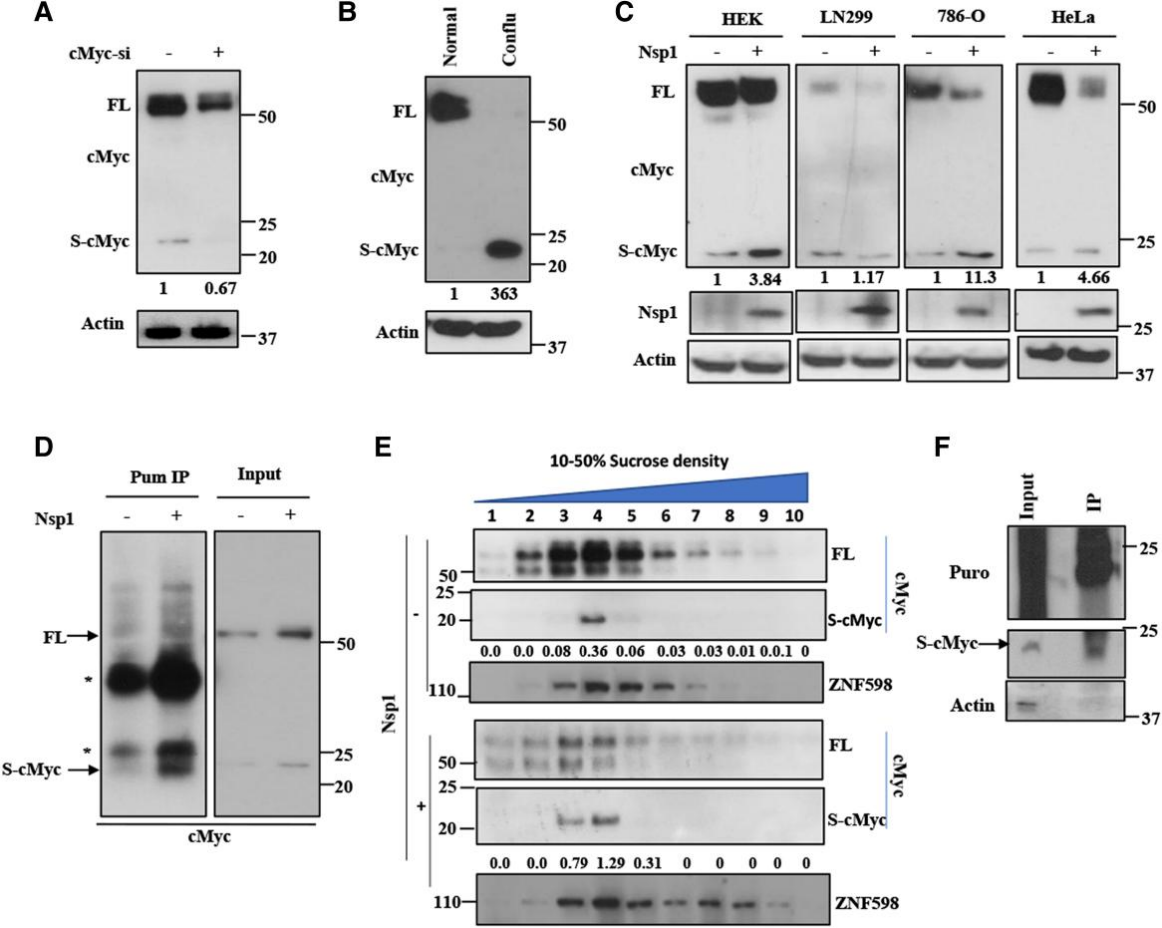
Translational stalling of cMyc. A) Immunoblots showing effect of cMyc RNAi on the levels of full-length (FL-) and short (S-) cMyc detected with the cMyc N-term antibody (D84C12). B) Immunoblots showing the effect of starvation and contact inhibition on FL-cMyc vs. S-cMyc level. C) Immunoblots showing the effect of Nsp1 on cMyc level and S-cMyc/FL-cMyc ratio in normal (HEK293) and cancer (HeLa, LN299, 786-O) cells. D) IP of Pum-labeled stalled nascent peptides followed by N-term cMyc antibody western blotting showing S-cMyc being a stalled nascent peptide. HeLa cells with or without Nsp1 transfection were treated with HHT (5 µM) for 10 min followed by emetine (100 µM) and Pum (100 µM) treatment for 15 min to label stalled peptides. Cells were subjected to IP with anti-Pum and immunoblotting with cMyc antibody (D84C12). Asterisks mark IgG light and heavy chains. 5× of input sample were used in IP assays. E) Immunoblots showing distribution of FL-cMyc and S-cMyc across sucrose gradient fractions of ribosomes from cell lysates pretreated with RNase. F) Immunoblots showing that collided ribosome-associated S-cMyc is labeled by Pum, supporting that it is a stalled NPC. Actin serves as a negative control. The values under cMyc immunoblots represent normalized ratios of S-cMyc/FL-cMyc in all panels.

We next sought to understand the mechanism by which Nsp1 down-regulates cMyc protein level. Our recent studies revealed a novel function of Nsp1 in regulating the translation of problematic host mRNAs that cause ribosome stalling (34). We first tested if *c-myc* translation encounters ribosome stalling. In HeLa cells, we detected a low abundance, smaller molecular weight (MW) species (∼20 kDa) of cMyc (S-cMyc) that was recognized by the cMyc antibody (D84C12) raised against residues surrounding Asp15 near the N-terminus of human cMyc (Fig. 2A), but not the cMyc antibody (E5Q6W) raised against more downstream amino acid sequence (Fig. S2A). The specificities of the D84C12 and E5Q6W antibodies were further confirmed by the ability of the former to recognize the N-terminal 1–170 but not C-terminal 170–439 fragment and the latter to recognize the C-terminal 170–439 but not N-terminal 1–170 fragment of cMyc (Fig. S2B). These data suggest that S-cMyc represents an N-terminal fragment of cMyc. This smaller cMyc species is distinct from the cMyc-S isoform resulting from the translation from a downstream AUG codon, which shares the C-terminus but not N-terminus with full-length (FL)-cMyc and has a higher MW (37). Both FL-cMyc and S-cMyc levels were reduced by cMyc RNAi, confirming antibody specificity and the notion that S-cMyc is related to cMyc (Fig. 2A). Although there exists another *myc* family member, B-Myc (38, 39), which is homologous to the N-terminal region of cMyc, S-cMyc is unlikely to be B-Myc for the following reasons: (ⅰ) B-Myc runs at 26 kDa on SDS-PAGE (40), whereas S-Myc runs slightly above 20 kDa; (ⅱ) B-Myc is so far only detected in rodents and in specific tissues (41), and our database searches did not find a human homolog of rodent B-Myc. Yet, S-cMyc is detected in human cells; (ⅲ) in rodents, B-Myc and cMyc sequences show significant divergence, making it unlikely for cMyc siRNA to knockdown B-Myc, whereas we observed that cMyc siRNA efficiently knocked down both FL-cMyc and S-cMyc; (ⅳ) even though *Drosophila* does not contain a B-Myc homolog, when mammalian cMyc was expressed in flies, S-cMyc was observed (Fig. S2C). Intriguingly, the abundance of S-cMyc was significantly increased relative to FL-cMyc in cells cultured for a longer time without medium change and was experiencing starvation and/or contact inhibition (Fig. 2B). In Nsp1-transfected cancer cells, FL-cMyc level was significantly reduced and the relative ratio of S-cMyc/FL-cMyc was significantly increased (Fig. 2C), suggesting that Nsp1 promotes the formation of S-cMyc, which also resulted in less synthesis of FL-cMyc. Though Nsp1 transfection in HEK293 cells also increased the S-cMyc/FL-cMyc ratio, its effect on FL-cMyc was limited (Fig. 2C), consistent with limited inhibition of HEK293 cell proliferation by Nsp1 and further suggesting that the antiproliferation effect of Nsp1 is rather specific to cancer cells. The presence of S-cMyc and increased S-cMyc/FL-cMyc ratio by Nsp1 were also observed in vivo in transgenic flies expressing mammalian cMyc (Fig. S2C). The reduction of both FL-cMyc and S-cMyc levels by Nsp1 in flies is analogous to the regulation of stalled APP.C99 translation we previously observed in the fly neuromuscular tissues (34), raising the possibility that *cMyc* translation is stalled in vivo.

To test whether S-cMyc is a product of stalled translation, we performed selective labeling of stalled NPCs with puromycin (Pum). The method involves a pretreatment of cells with homoharringtonine (HHT), which blocks formation of the first peptide bond but allows already initiated, actively elongating ribosomes to run off. This is followed by a combined emetine and Pum treatment to incorporate the tRNA-like Pum to the very C-termini of stalled NPCs that remain after the running off of active ribosomes (42). This method thus specifically labels stalled NPCs with Pum. After IP with anti-Pum to pull down stalled NPCs followed by probing with the N-term cMyc antibody (D84C12), we found that S-cMyc was Pum labeled (Fig. 2D), supporting that it is a stalled NPC. Anisomycin is a translation elongation inhibitor and well known for its ribosome stalling activity (43). Anisomycin treatment significantly increased the S-cMyc/FL-cMyc ratio (Fig. S2D), consistent with S-cMyc being a stalled translation product of cMyc.

A prerequisite for S-cMyc being a stalled NPC is its association with stalled ribosomes. To test whether S-cMyc is associated with stalled ribosomes, we performed sucrose gradient fractionation experiments. Treatment of cell lysates with RNase, which is commonly used to collapse translating polysomes into monosomes but leave collided ribosomes intact due to the resistance of disome-covered mRNA to RNase treatment (44, 45), showed that S-cMyc was present in fractions 4 and 5 (Fig. 2E), in which ZNF598 was also enriched. As ZNF598 is a sensor of ribosome collisions and enriched in collided ribosomes, this result supported that S-cMyc was associated with stalled/collided ribosomes. Moreover, the ratio of S-cMyc/FL-cMyc in fractions 3–5 was significantly increased in Nsp1 co-expression condition (Fig. 2E), consistent with Nsp1 promoting the translation stalling of cMyc. To further confirm that S-cMyc present in fraction 4 was indeed a stalled translation product, we performed sucrose gradient fractionation of lysates from cells subjected to Pum labeling of stalled NPCs. We immunoprecipitated Pum-labeled proteins from fraction 4 with the anti-Pum antibody and probed it with anti-cMyc antibody (D84C12). S-cMyc present in fraction 4 was found to be specifically puromycinylated (Fig. 2F), supporting that it is a stalled NPC.

### The RQC pathway critically regulates stalled translation of cMyc

**Fig. 3.**
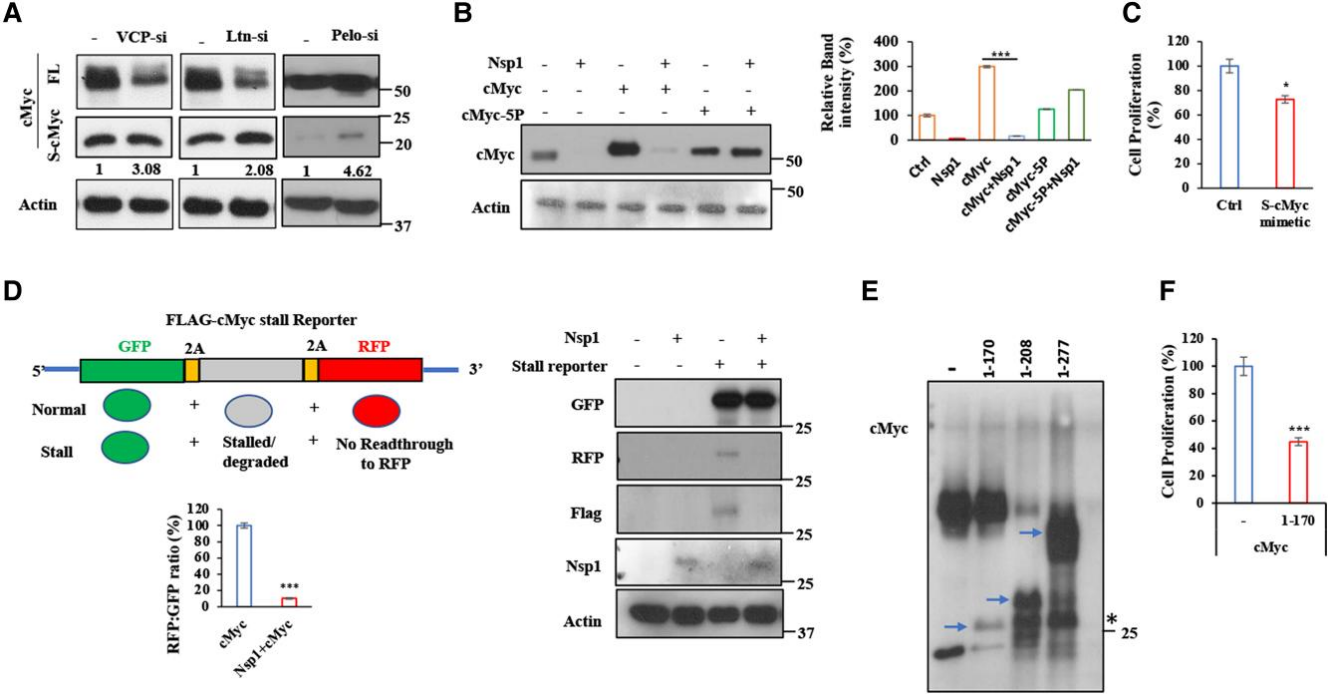
Effect of RQC factors and Nsp1 on stalled Myc translation and mapping of the stall site. A) Immunoblots showing effect of RNAi of RQC factors VCP, Ltn, and Pelo on FL- and S-cMyc protein levels. The relative ratio of S-cMyc/FL-cMyc was measured and indicated with values under the cMyc blots. B) Immunoblots showing effect of the Pro–Pro motif mutations in cMyc on cMyc level in HeLa cells with or without the co-expression of Nsp1. C) Effect of S-cMyc mimicking cMyc N-term domain construct on HeLa cell proliferation in the MTT assay. D) Diagram of the Flag-cMyc stall reporter and measurement of cMyc translation stalling by RFP/GFP ratio. Immunoblots show the effect of Nsp1 on GFP-P2A-Flag-S-cMyc-P2A-RFP reporter expression in HeLa cells. E) Immunoblots showing FL (arrows) and stalled translation products (*) expressed from HA-cMyc1-170, HA-cMyc1-208, and HA-cMyc1-277 constructs. Note that the stalled proteins from HA-cMyc1-208 and HA-cMyc1-277 constructs were of similar size as that of HA-cMyc1-170. F) MTT assay showing toxicity of HA-cMyc1-170 to HeLa cells. The results were quantified by NIH ImageJ software and statistically analyzed by GraphPad Prism software (***P < 0.001, **P < 0.01, *P < 0.05 in Student's t tests or one-way ANOVA test followed by Student–Newman–Keuls posttest).

The RQC pathway regulates the translation of stalled mRNAs by surveying incomplete NPCs produced by stalled ribosomes and targeting them for degradation (11). Three genes critically involved in the quality control of stalled translation are Ltn1, VCP, and Pelo. We found that RNAi-mediated knockdown of Ltn1, VCP and Pelo with validated siRNAs resulted in significantly increased abundance of S-cMyc relative to FL-cMyc (Fig. 3A). This robust response of S-cMyc abundance to the genetic manipulation of three distinct RQC factors provided strong support that it represents stalled cMyc species. Although it has been conjectured that ribosomes pause during the translation elongation of cMyc (46, 47), *bona fide* stalled translation product of cMyc has not been identified before this study, presumably because stalled NPCs are transient species that are subject to degradation by the quality control systems and their detection depends on antibody sensitivity, the particular cell lines used, and cell growth conditions. Recent ribosome profiling studies of collided ribosomes (disomes) in mammalian cells identified Pro–Pro motifs as putative ribosome pausing motifs (44, 45). In the N-terminal region of cMyc, there were five such motifs that have been implicated in influencing the translation elongation of cMyc (47). We found that whereas cMyc-WT level was dramatically reduced when co-transfected with Nsp1, the level of a mutant cMyc with the potential stall-inducing PP motifs mutated to AA (cMyc-5P) was relatively immune to Nsp1 action (Fig. 3B), consistent with Nsp1 regulating stalled translation of cMyc. The resistance of cMyc-5P to Nsp1 action was unlikely due to unintended effect of the 5P mutations on cMyc stability, as our time course assessment of protein level in cells treated with cycloheximide to inhibit new protein synthesis did not reveal obvious difference between cMyc-WT and cMyc-5P (Fig. S3A), suggesting that the 5P mutations did not affect cMyc stability.

Based on MW, S-cMyc likely encompasses the transactivation and transrepression domain containing Myc box I (MBI), MBII, and MBIII. We wondered if S-cMyc resulting from translation stalling possess any biological activity. When the N-terminal 200 amino acids of cMyc approximating S-cMyc was expressed in HeLa cells, it resulted in growth inhibition (Fig. 3C), accompanied by changes in signaling molecule (p-mTOR and ZNF598) levels (Fig. S3B), suggesting that S-cMyc may inhibit cell growth. This is consistent with the N-terminal region of cMyc possessing growth inhibition activity (39). We next generated a GFP-P2A-Flag-S-cMyc-P2A-RFP reporter to assess the effect of Nsp1 on ribosome stalling during S-cMyc translation. In this reporter, the GFP, Flag-tagged S-cMyc mimetic (1–200), and RFP reporters are used to monitor overall mRNA translation, translational stalling at S-cMyc, and read-through of the stall, respectively, with the self-cleaving P2A releasing each reporter and allowing them to be independent marker of translation. Nsp1 co-transfection significantly reduced the ratio of RFP/GFP expressed from the reporter (Fig. 3D), suggesting that read-through of the cMyc stall site was blocked by Nsp1.

To more precisely map the stall site that leads to S-cMyc, we expressed three HA-tagged cMyc constructs expressing the 1–170, 1–208, and 1–277 fragments of cMyc, respectively. We found that while the cMyc (1–170) construct produced a single peptide (HA-cMyc1–170), c-Myc (1–208), and cMyc (1–277) each produced a common shorter peptide running at similar position as HA-cMyc1–170 and another peptide that corresponded to FL HA-cMyc1–208 or HA-cMyc1–277, respectively (Fig. 3E). This result suggested that both 1–208 and 1–277 fragments of cMyc encompassed the translation stall site, and the stall site is very close to amino acid 170. When HA-cMyc1–170 was expressed in HeLa cells, it inhibited cell growth in MTT assays (Fig. 3F), consistent with our earlier finding with cMyc (1–200) (Fig. 3C) and the reported growth-inhibiting activity of this N-terminal portion of cMyc (39).

### Nsp1 interacts with the RQC machinery and co-opts ABCE1 to down-regulate cMyc

**Fig. 4.**
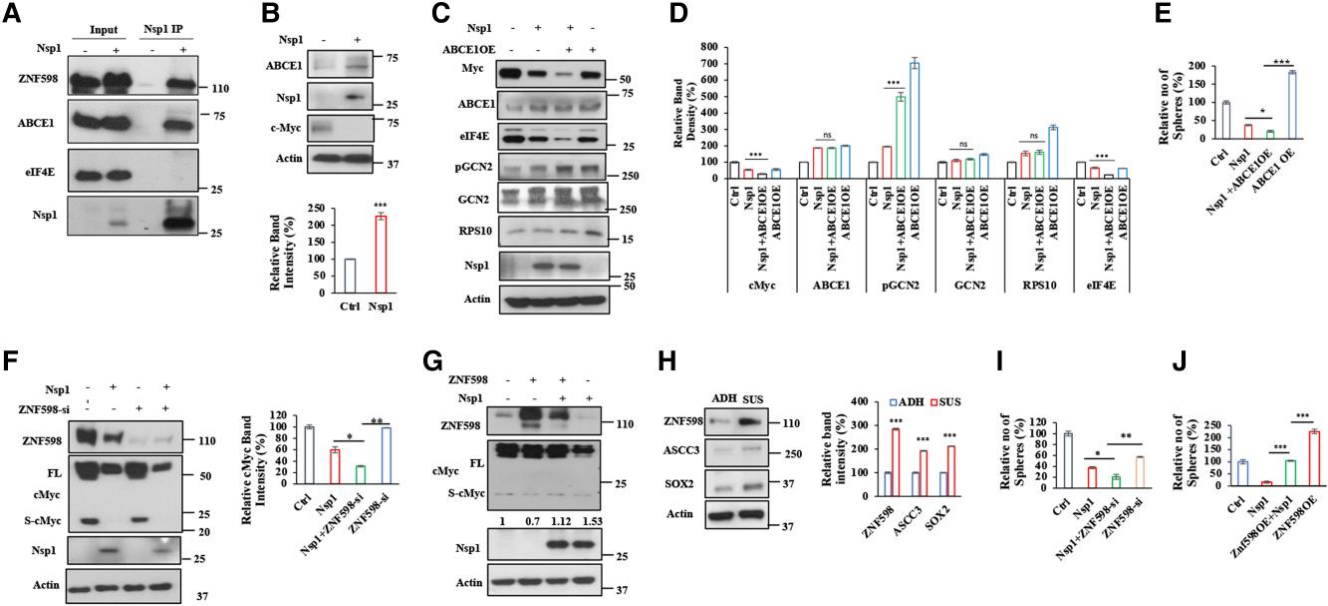
Effects of ZNF598 and the RQC pathway on cMyc translation and cancer cell growth. A) Co-IP assays showing Nsp1 interaction with ABCE1 and early RQC factor ZNF598 and ASCC3, but not the translation initiation factor eIF4E. 5× of input sample was used in co-IP assays. B) Immunoblots and quantification showing effect of Nsp1 on ABCE1 and cMyc protein levels. C, D) Immunoblots (C) and quantification (D) showing effect of Nsp1 and ABCE1 co-expression on cMyc and other protein levels. E) Effect of Nsp1 and ABCE1 co-expression on GBM neurosphere formation. F, G) Effect of ZNF598 knockdown (F) or OE (G) on cMyc protein level. Values under cMyc blot in (G) indicate relative S-cMyc/FL-cMyc ratio. H) Immunoblots and quantification of levels of the RQC and stemness factors in adherent (ADH) vs. suspension (SUS) LN299 GBM cells. I, J) Effect of ZNF598 RNAi (I) and OE (J) on GBM neurosphere formation with or without Nsp1 co-expression. Results were quantified by NIH ImageJ software and statistically analyzed by GraphPad Prism software (***P < 0.001, **P < 0.01, *P < 0.05 in Student's t tests or one-way ANOVA test followed by Student–Newman–Keuls posttest).

We next investigated the molecular mechanisms by which Nsp1 regulates the translation stalling of cMyc. The ribosome splitting and recycling factor ABCE1 were previously shown to interact with Nsp1 (29) and participate in the handling of stalled translation of APP.C99 (34). We found that ABCE1 interacted with Nsp1 in cancer cells as detected by co-immunoprecipitation (co-IP) assay (Fig. 4A). Interestingly, unlike cMyc, ABCE1 protein level was increased in Nsp1-transfected cells (Fig. 4B), suggesting that Nsp1 does not indiscriminately repress translation or that the physical interaction with Nsp1 may serve to stabilize ABCE1. Remarkably, ABCE1 co-expression enhanced the effect of Nsp1 in down-regulating cMyc protein level and inhibiting GBM neurosphere formation (Fig. 4C, D). This synergy between Nsp1 and ABCE1 in down-regulating cMyc was not simply due to inhibition of cell growth, as the levels of several other proteins examined, including Rps10, Actin, GCN2, and p-GCN2, were not reduced by the co-expression of Nsp1 and ABCE1 as in the case of cMyc (Fig. 4E). On the other hand, ABCE1 RNAi did not significantly affect the growth inhibition of GBM cells by Nsp1 (Fig. S4A), suggesting that ABCE1 facilitates Nsp1 function but it is not the only player mediating Nsp1 action, consistent with Nsp1 employing multipronged strategies to manipulate host cell function (48, 49).

The synergy between Nsp1 and ABCE1 in down-regulating cMyc and inhibiting cancer cell growth is unexpected, as ABCE1 tends to be up-regulated in cancer cells (Fig. S4B) and its supporting role in oncogenesis has been observed in multiple cancer settings (50, 51). Indeed, we found that cMyc overexpression (OE) up-regulated the expression of ABCE1 (Fig. S4C), consistent with cMyc being a master regulator of ribosomal proteins and translation factors. Our analysis of human cancer gene expression in Pan-cancer analysis of whole genomes (ICGC/TCGA) (52) revealed that the expression of c-Myc positively correlated with that of ABCE1 (Fig. S4D). Thus, while ABCE1 is a potential downstream target of cMyc and may positively participate in Myc-driven cancer growth (50), it is co-opted by Nsp1 to inhibit cMyc translation and cancer growth, making cMyc overexpressing cancer cells with up-regulated ABCE1 protein level particularly vulnerable to the effect of Nsp1.

We performed further co-IP studies to identify other RQC factors that may interact with Nsp1 and mediate its effect on cMyc translation. We found that Nsp1 interacted with ZNF598, which recognizes the distinct 40S–40S interface characterizing collided ribosomes and promotes the ubiquitination of specific 40S subunit proteins (17, 43), and ASCC3, a key component of the ASC complex that disassembles stalled ribosomes (18) (Fig. 4A). Both ZNF598 and ASCC3 are early RQC factors. However, unlike ABCE1, ZNF598 and ASCC3 levels were reduced by Nsp1 (Figs. 1C and 4F). These data raised the possibility that reduction of ZNF598 level may present yet another mechanism through which Nsp1 regulates cMyc translation stalling, as ZNF598 senses ribosome collision and initiates activation of downstream RQC processes to handle stalled ribosomes. Consistent with this possibility, ZNF598 RNAi further reduced FL-cMyc level in Nsp1-transfected cells (Fig. 4F). On the other hand, ZNF598 OE decreased the level of S-cMyc and the ratio of S-cMyc/FL-cMyc in Nsp1-transfected cells (Fig. 4G).

### The early RQC factor ZNF598 is up-regulated in GBM cancer stem cells and promotes cancer stem cell growth

We next sought to test the role of ZNF-598 in Myc-driven cancer growth. We first used the GBM model. It is known that growth conditions can affect the properties of GBM cancer stem cells (CSCs) (53). We found that compared with adherent culture (2D), GBM cells grown in suspension as spheres (3D) expressed higher levels of the stem cell factor Sox2 (Fig. 4H), suggesting that GBMs in suspension culture are enriched for CSCs and presumably more malignant. Interestingly, the early RQC factors ZNF598 and ASCC3 were both up-regulated when GBM cells were in 3D suspension culture (Fig. 4H), suggesting that the RQC pathway is more active in GBM CSCs. Given the link between RQC and cMyc translation and the essential role of ZNF598 in RQC, we examined the effect of ZNF598 on cMyc-driven cell growth. We found that OE of ZNF598 increased nucleolar size (Fig. S4E) and that cMyc and its known target eIF4E levels were significantly increased by ZNF598 (Fig. S4F), consistent with cMyc being a target of ZNF598 regulation. To assess the functional role of ZNF598 on CSC behavior, we tested the effect of ZNF598 OE and RNAi on GBM CSC self-renewal in neurosphere assays. ZNF598 knockdown significantly decreased neurosphere formation on its own and further enhanced the Nsp1 effect, whereas ZNF598 OE had opposite effects (Fig. 4I, J). These results suggest that ZNF598 and Nsp1 exert opposite effects in regulating CSC homeostasis, presumably through their antagonistic effects on the translational QC of cMyc.

### Effect of ZNF598 and the RQC pathway in regulating Myc-dependent cancer growth in vivo

**Fig. 5.**
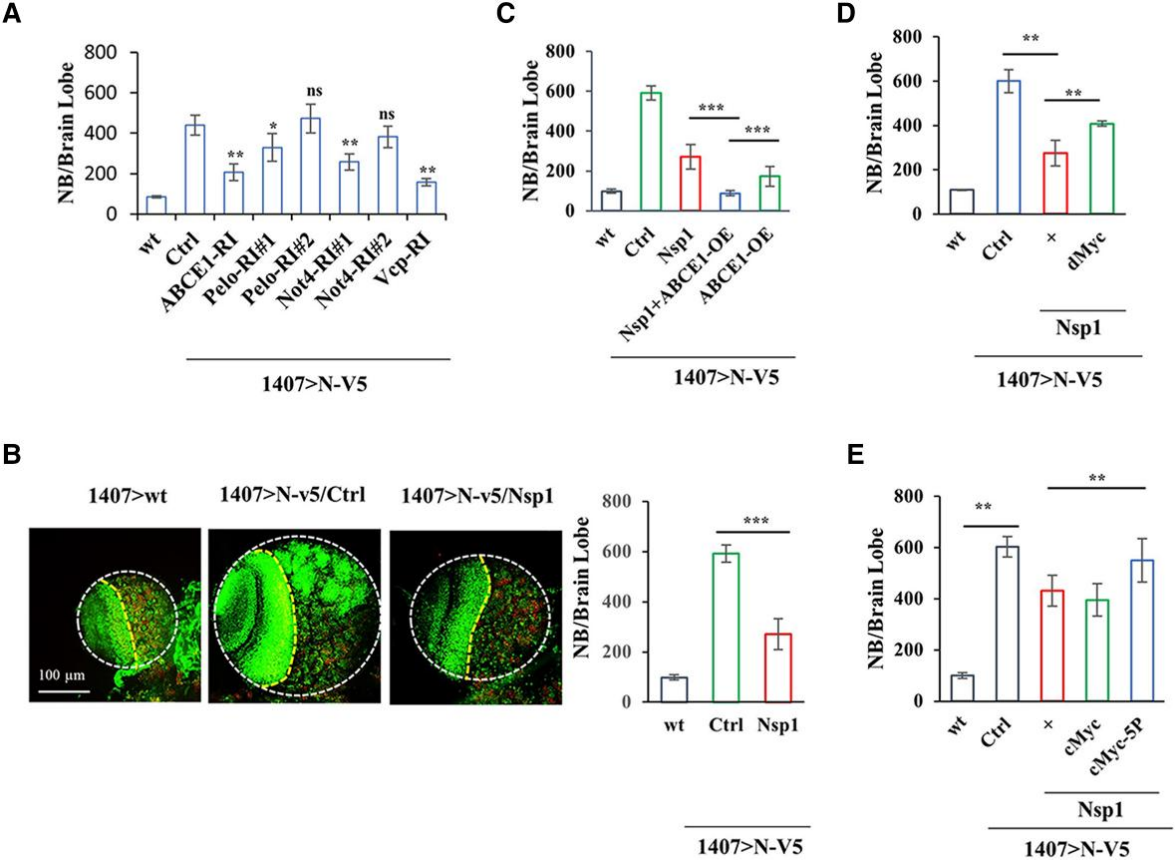
Effects of the RQC pathway in regulating Myc translation and Myc-dependent tumor growth in vivo. A) Effect of genetic manipulation of RQC factors ABCE1, Pelo, Not4, and VCP on Notch-induced brain tumor phenotype in *Drosophila* larval brain. B) Immunostainings and data quantification showing effect of Nsp1 on Notch-induced brain tumor phenotype. Larval brains were immunostained with the NB marker Dpn and differentiation marker Pros. Larval brains were outlined with white dotted lines. The yellow dotted lines separate the optic lobe (left) from the central brain (right). C) Effect of ABCE1 and Nsp1 co-expression on Notch-induced brain tumor phenotype in *Drosophila* larval brain. D) Effect of dMyc OE in attenuating the inhibitory effect of Nsp1 on Notch-induced brain tumor phenotype. E) Effect of cMyc and cMyc-5P in attenuating the inhibitory effect of Nsp1 on Notch-induced brain tumor phenotype. Results were quantified by NIH ImageJ software and statistically analyzed by GraphPad Prism software (***P < 0.001, **P < 0.01, *P < 0.05 in Student's t tests or one-way ANOVA test followed by Student–Newman–Keuls posttest).

Next, we tested the significance of the RQC pathway on cancer behavior. To examine in vivo effects, we used a Notch-induced brain tumor model in *Drosophila*, which is particularly dependent on dMyc function (54). We first tested the involvement of ribosome-associated co-translational quality control factors in neural stem cell (NSC) and CSC homeostasis. We found that knockdown of the RQC-related genes *Pelo*, *ABCE1*, and *NOT4* with validated RNAi tools all attenuated Notch-induced overproliferation of larval neuroblasts (NBs), the NSCs in the fly brain (Figs. 5A and S5A), and rescued animal survival from pupal lethal to viable adults. Conversely, Pelo OE showed slight enhancement of Notch-induced NB overproliferation (Figs. 5A and S5G). CSC-like brain tumor-forming cells apparently are particularly sensitive to perturbation of these co-translational QC factors, as their knockdown blocked Notch-induced CSC-like NB overproliferation without affecting normal NB maintenance. Nevertheless, complete loss of *Pelo* impaired intermediate progenitor (IP) proliferation in a wild-type background (Fig. S5B). These results indicate a critical role of co-translational QC in regulating NSC homeostasis under both normal and brain tumor conditions, although the stringency of requirement for individual factors may differ. The RNAi of VCP and Listerin (Ltn), homologs of key components of the yeast RQC machinery, both attenuated NSC over-proliferation induced by Notch OE (Figs. 5A and S5A–D, G). Again, CSC-like NBs were more sensitive than normal NBs to the knockdown of these RQC factors. In the case of VCP, loss-of-function in wild-type condition also affected NB and IP proliferation (Fig. S5E), an effect seen in Notch RNAi condition (54). This is consistent with the finding of VCP being a direct substrate of a noncanonical Notch signaling pathway involving PINK1 and mTORC2/AKT (28), which is particularly relied upon by CSC-like NBs (55). Together, these data support a critical role of the RQC pathway in regulating NSC homeostasis under normal and brain tumor conditions, with CSC-like aberrant NBs being more dependent on this pathway.

Myc is a regulator of NSC growth and indispensable in Notch-induced dedifferentiation from IPs to CSC-like NBs (54, 56). We tested whether, by down-regulating Myc, Nsp1 would affect Notch-induced CSC-like NB overgrowth. Indeed, Nsp1 partially attenuated Notch-induced brain tumor phenotype (Fig. 5B). ABCE1 OE also partially rescued Notch-induced brain tumor phenotype, and Nsp1/ABCE1 co-expression, which synergistically down-regulated dMyc translation as in cMyc case in mammalian cells, resulted in a more complete inhibition of Notch-induced NB overgrowth (Figs. 5C and S5F, G). Consistent with dMyc being a key target of Nsp1 in this process, its OE showed a partial blockage of the Nsp1 effect (Figs. 5D and S5H). In line with ribosome stalling during cMyc translation being an impediment to cMyc translation and cMyc-driven cell growth, we found that while cMyc-WT did not affect total brain NB number, the ribosome stalling-resistant cMyc-5P mutant resulted in mild NB overproliferation when ectopically expressed (Figs. 5E and S5H, I). The differential effects of cMyc-WT and cMyc-5P were unlikely caused by differential expression of the transgenes, as the transgenes were inserted into the same genomic locus using the PhiC31 integrase-mediated transgenesis system, and their mRNA expression was comparable (Fig. S5J). Importantly, while cMyc-WT failed to attenuate the Nsp1 effect on Notch-induced NB overproliferation, presumably due to its sensitivity to Nsp1 regulation, cMyc-5P significantly reverted the Nsp1 effect (Figs. 5E and S5H, I), supporting that regulation of stalled *c-myc* translation is a key mechanism by which Nsp1 regulates CSC growth.

**Fig. 6.**
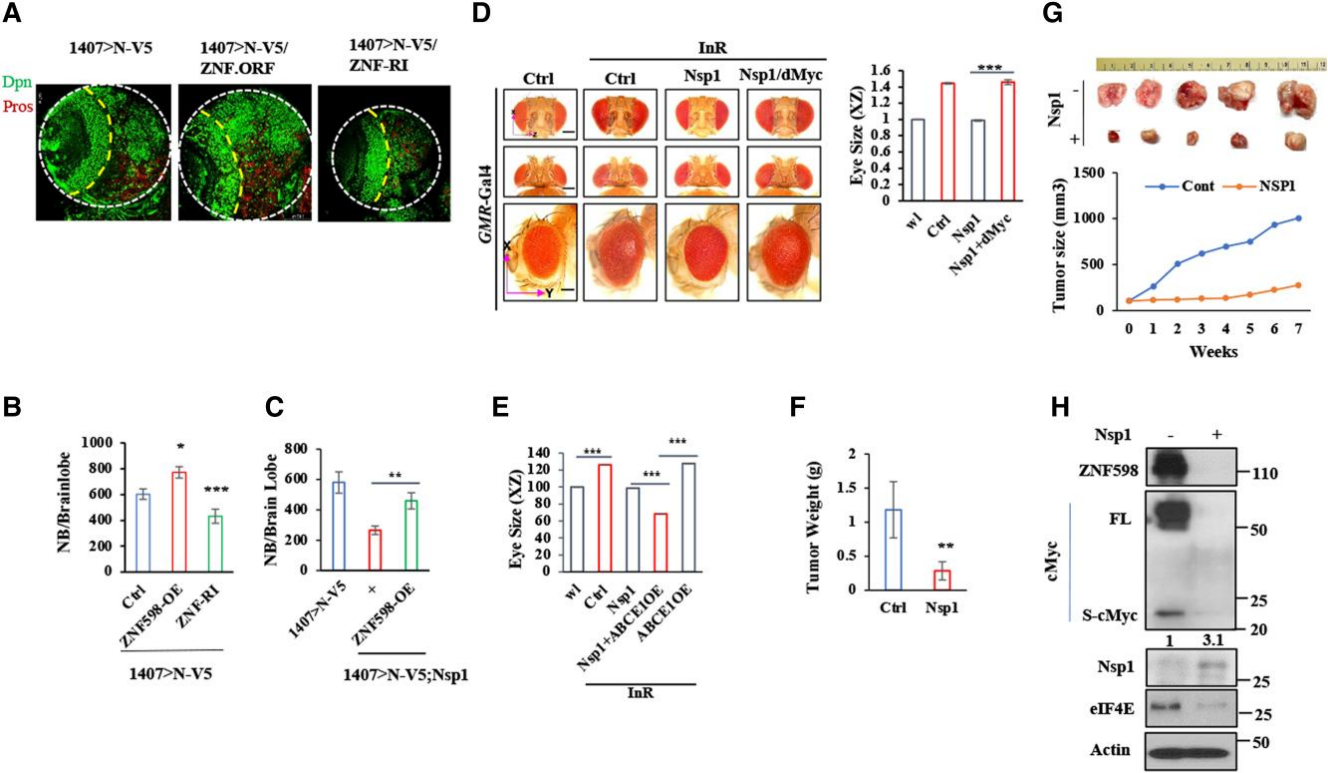
Effect of ZNF598 on Notch-induced brain tumor in *Drosophila* and Nsp1 on xenograft tumor growth in mouse. A, B) Images (A) and data quantification (B) showing effect of genetic manipulation of ZNF598 activity on Notch-induced brain tumor phenotype. C) Effect of ZNF598 OE in attenuating the inhibitory effect of Nsp1 on Notch-induced brain tumor phenotype. Images are shown in Fig. S6C. D) Images and data quantification showing effect of dMyc co-expression in attenuating the inhibitory effect of Nsp1 on InR-induced overgrowth phenotype in the fly eye. E) Effect of ABCE1 and Nsp1 co-expression on InR-induced eye overgrowth phenotype. F) Effect of intratumoral AAV-Nsp1 injection on tumor weight (g) in a xenograft tumor model in BALB/c pathogen-free athymic nude mice after 7 weeks. G) Images of final tumor mass and quantification of tumor volume showing effect of intratumoral delivery of AAV-Nsp1 on tumor growth. H) Immunoblots showing effect of Nsp1 on cMyc, ZNF598, and eIF4E levels in tumor tissues collected at end of study. Values under cMyc blot indicate relative S-cMyc/FL-cMyc ratio. Results were quantified by NIH ImageJ software and statistically analyzed by GraphPad Prism software (***P < 0.001, **P < 0.01, *P < 0.05 in Student's t tests or one-way ANOVA test followed by Student–Newman–Keuls posttest).

To examine the in vivo effect of ZNF598 on NSC homeostasis, we tested the effect of ZNF598 OE and RNAi under normal or in Notch-induced brain tumor conditions. ZNF598 OE and RNAi had no obvious effect on total NB number or brain size under otherwise normal condition (Fig. S6A). In Notch-induced brain tumor condition, however, ZNF598 protein expression was up-regulated (Fig. S6B), and ZNF598 RNAi significantly reduced NB overgrowth, whereas ZNF598 OE showed slight enhancement (Fig. 6A, B). Moreover, ZNF598 OE attenuated the inhibitory effect of Nsp1 on dMyc expression and Notch-induced NB overgrowth (Figs. 6C and S6C, D). These data, therefore, support ZNF598 being a critical player in CSC proliferation and growth regulated by Myc and Notch signaling.

We also tested the effect of Nsp1 on cancer cell growth in other settings that also involve Myc. Hyperactivation of the insulin receptor (InR)/PI3K/AKT, Rheb/mTOR, Ras, or Yorki pathways all lead to overgrowth of the fly eye. These cell growth pathways all depend on dMyc (57–60). Nsp1 co-expression restored fly eyes to normal sizes, and this effect was blocked by the co-expression of dMyc (Figs. 6D and S6E). As in Notch-induced brain tumor condition, co-expression of Nsp1 and ABCE1 further enhanced the Nsp1 effect in inhibiting InR-induced eye overgrowth. These results further support the co-opting of ABCE1 by Nsp1 in its anticancer action (Fig. 6E). Furthermore, compared with cMyc-WT, cMyc-5P was more potent in inducing eye overgrowth (Fig. S7A) and in suppressing the antigrowth effect of Nsp1 (Fig. S7B, C). These data support the notion that Nsp1 acts through the RQC of stalled Myc translation to restrain Myc-driven cell growth in cancer. As observed in cultured cells, the co-expression of Nsp1 with ABCE1 resulted in a more dramatic reduction of dMyc level, and eye growth was inhibited in both normal condition and InR-induced overgrowth condition, resulting in smaller than normal eyes (Fig. S8A, B).

To further test the conservation and therapeutic potential of inhibiting cMyc-driven tumor growth by Nsp1, we generated a mouse xenograft tumor model by subcutaneous injection of HeLa cells into nude mice. When tumors reach 100 mm^3^ in size, AAV-Nsp1 was injected intratumorally. Nsp1 expression did not change animal body weight or overall health, but significantly blunted tumor growth (Fig. 6F, G). This was correlated with reduced cMyc and ZNF598 proteins levels and increased cMyc stalling as indicated by the increased S-cMyc/FL-cMyc ratio, and reduced cMyc target eIF4E level (Fig. 6H), supporting the relevance of the RQC of stalled cMyc translation in a mammalian cancer setting.

## Discussion

RQC is emerging as an important mechanism guarding the integrity and fidelity of the proteome. Relative to the ribosomes, the RQC factors are present at substoichiometric levels, and their deficiency under stress or aging conditions can lead to failures in the maintenance of the cellular proteome and contribute to age-related neurodegenerative diseases. Despite the importance of translational control to cancer cell growth, proliferation, and differentiation, the role of RQC in cancer biology has been underexplored. In this study, we present evidence that the master cell growth regulator Myc represents a key RQC substrate in NSC and CSCs. This finding offers new avenues for elucidating the in vivo function of RQC, and potential new strategies to target the “undruggable” cMyc.

We demonstrate that ribosomes stall during the translation of the N-terminus of cMyc, resulting in the generation of stalled S-cMyc. This conclusion is supported by the following evidence: (ⅰ) S-cMyc is labeled by Pum using a protocol that selectively labels stalled NPCs; (ⅱ) the relative abundance of S-cMyc is directly regulated by key genes in the RQC pathway that handle stalled translation; (ⅲ) the abundance of S-cMyc is regulated by Nsp1 that also manipulates stalled translation; (ⅳ) the abundance of S-cMyc is influenced by treatment with anisomycin under conditions that induce ribosome stalling; (ⅴ) puromycin-labeled S-cMyc is associated with collided ribosomes in sucrose gradient fractionation assays; (ⅵ) the cMyc-5P mutant with putative translation stall signal altered exhibited differential in vivo effects and responses to stall inducing conditions compared with cMyc-WT in a manner unaccounted for by differential protein stability, consistent with their differential regulation by ribosome stalling. The presence of translation stall in Myc is likely to serve a physiological role rather than simply being an idiosyncrasy of c-Myc protein. As Myc hyperactivity in proliferating cells can lead to cancer, and excessive Myc expression can lead to apoptosis (61), tight control of Myc protein level will be a key to NSC homeostasis. Indeed, previous studies implicated a critical role of translational or posttranslational control of Myc protein expression in NSCs (54, 62). Thus, despite previous studies implicating Myc as a transcriptional target of Notch signaling (54, 63), and mammalian studies showing c-Myc OE being sufficient to lead to neoplastic transformation under certain conditions (64), transcriptional up-regulation of dMyc is insufficient to recapitulate the effect of Notch in inducing ectopic NB formation in flies (54), where Myc protein level remaining low despite elevated mRNA level (62). This suggests that there exists tight homeostatic regulation of Myc protein level in NSCs. We hypothesize that in proliferating stem cells or cancer cells, which feature increased protein synthesis and pose increased risk for ribosome collisions, the ribosome stalling and subsequent RQC may serve as a negative feedback loop to control Myc protein level, maintain stem cell homeostasis and prevent apoptosis.

Our results show that ribosome stalling during heightened cMyc translation and the up-regulated expression of translation factors such as ABCE1 by cMyc present vulnerabilities in cMyc-driven cancers that can be targeted by Nsp1 through the ZNF598 RQC pathway. As a novel therapeutic tool, Nsp1 acts through at least two mechanisms to perturb cMyc-driven cancer growth: inhibition of cMyc expression, and synthetic lethality with genes up-regulated by cMyc (e.g. ABCE1). While further studies are needed to better understand the mechanisms of action of Nsp1 and the normal role of Nsp1 in handling stalled ribosomes in the context of viral replication and viral-host interaction, our current data suggest that Nsp1 offers a potential therapeutic agent derived from SARS-Cov-2 that can be leveraged for treating many types of human cancers driven by cMyc. Further studies will test whether RQC of stalled *cMyc* translation can be exploited for effective cancer therapy.

## Materials and methods

### Cells and culture conditions

The normal fibroblasts, HEK293, LN299, and HeLa cells were obtained from American Tissue Culture Collection and cultured in DMEM while 786-O cells were cultured in RMPI1640 media. Each medium was supplemented with 10% fetal bovine serum (FBS), 2 mmol/L l-glutamine, 100 U/mL penicillin, and 100 mg/mL streptomycin.

GBM-387 and 3691 cells were previously published cell lines provided by Dr Siddhartha Mitra from Sam Cheshire's Lab. GBM cells were cultured in neurobasal medium containing B27 without retinoic acid, human-bFGF (20 ng/mL), human-EGF (20 ng/mL), and heparin (10 ng/mL). Neurospheres were maintained in serum-free media, while neurospheres were differentiated into monolayer adherent cells by growing them in DMEM supplemented with 10% FBS for 20–30 days.

For proliferating vs. confluent culture conditions that induce S-cMyc, HeLa cells were grown up to confluent condition and maintained in confluent condition at least 24 h, then cells were collected and processed for further experiments. For siRNA knockdown experiments, cells were transfected with siVCP (Invitrogen HSS123962), siListerin (Invitrogen 134567), and siPelo (Invitrogen 131910) using lipofectamine 2000 as transfection agent according to instructions from the manufacturers. For anisomycin treatment condition, HeLa cells were treated with varying concentrations of anisomycin for 1 h at 37 °C. After incubation, cells were collected and processed for further experiments.

### qRT-PCR

Total RNA was isolated using Trizol and cDNA was immediately synthesized using iScript cDNA synthesis kits (Bio-Rad). Thereafter, qPCR was performed using SYBER green master mix (A25741, Bio-Rad). The relative fold change was obtained by following the ΔΔCt method. Primer sequences were described in oligonucleotide section. Statistical significance of fold change was measured using GraphPad.

### Cell viability assay

Cell viability was measured by MTT bioassay. Briefly, treated cells were incubated with MTT (5 mg/mL in phosphate buffered saline (PBS)) for an additional 4 h. The dark-blue formazan crystals formed in cells were dissolved in DMSO, after which the absorbance at 570 nm was measured using an ELISA plate reader (Bio-Tek Instrument Co, Winooski, VT, USA). Cell viability assays were generally performed at 72–96 h after transfection.

### Lentivirus preparation and transfection

To overexpress Nsp1 in sphere cells, lentivirus of Nsp1 were produced in HEK293T cells using Lentivirus packaging vectors viz. pMDLg/pRRE, CMV-VSVG, and RSV-Rev. Then, lentivirus containing media was concentrated with Lenti-X Concentrator (Clontech Laboratories, #631231) and stored in −80 °C. These concentrated lentivrus were transfected in the cells by using polybrene to enhance the transfection efficiency. Cells were collected for further experimentation.

### Transfection of cells

Gene manipulation was achieved by using small interference RNA (siRNA), small hairpin (sh) RNA (shRNA), and OE plasmids. Transfection was performed using the lipofectamine 2000 reagent according to the manufacturer's instruction. Briefly, cells were transfected with a well-optimized mixture containing siRNA/shRNA/plasmid, transfection reagent, and Opti-MEM-reduced serum media then incubated at 37 °C. Cells were collected and processed for further experimentation. For western blot analysis, cells were collected before signs of cell death, generally 24–48 h after transfection. See Table S2 for a list of shRNAs, siRNAs, and plasmids used, and Table S3 for a list of chemicals used.

### Western blot analysis on fly tissues

Treated cells and dissected *Drosophila* tissues were harvested and lysed with radio-immunoprecipitation assay (RIPA) lysis buffer (Sigma-Aldrich, cat. R0278). Samples were resolved on NuPAGE 4–12% Bis-Tris Protein Gels (cat#: NP0321BOX, Invitrogen) and ran in MOPS SDS running buffer (cat#: NP0001, Invitrogen) and electro-transferred onto 0.2 µm polyvinylidene fluoride membrane (Roche Diagnostics, Indianapolis, IN, USA). Blots were then probed overnight with primary antibodies (Table S1, List of antibodies) followed by horseradish peroxidase-conjugated secondary antibody. Finally, membrane development was conducted using enhanced chemiluminescence and analyzed by X-ray film development.

### Immunocytochemistry

Treated cells were fixed using 4% paraformaldehyde for 15 min at room temperature followed by thrice washing with PBS for 5 min, then permeabilized by PBS containing 0.2% Triton X-100 with subsequent thrice washing with PBS. Then, cells were blocked using 1% bovine serum albumin containing 0.02% Tween 20 for 30 min at 37 °C followed by overnight incubation with primary antibody. The next day, cells were washed three times with PBS with 0.02% Tween 20 (PBST) and incubated with fluorescently labeled secondary antibodies for 1 h at room temperature with subsequent thrice washing with PBST and counterstained with mounting media with DAPI. Finally, labeled signals were observed using a Leica SP8 Confocal Microscope at 400× magnification.

### Translation stalling reporter assay

The GFP-P2A-Flag-S-cMyc-P2A-RFP reporter containing 3xFlag tagged N-terminal 200 amino acids of cMyc flanked by GFP and RFP with P2A sequence in between was synthesized at GenScript and sequence confirmed. Analysis of translation read-through at cMyc N-Term sequences using the stall reporter construct GFP-P2A-Flag-S-cMyc-P2A-RFP was performed as previously described (34). Briefly, HeLa cells were co-transfected with the reporter plasmid with or without Nsp1 plasmid co-transfection for 48 h. Cells were lysed and processed for western blot assay. Reporter read-through was measured by RFP/GFP ratio.

### Co-IP assay

Cells lysates were processed in NP40 IP-lysis buffer (5 M NaCl, 10% NP-40, 1 M Tris (pH 8.0)) with protease inhibitor cocktail (cat#: 11836170001, Sigma) added. After centrifugation at 10,000 rpm for 10 min, the supernatant was subjected to incubation with primary antibodies at 4°C overnight with gentle shaking. Subsequently, the A/G agarose beads were added for further 2 h incubation and thereafter washed three times (10 min each) with spinning at 1,000 rpm/4 °C in PBS. Samples were then boiled at 97°C for 5 min with 2× loading dye. Samples were analyzed by western blotting.

### Clonogenicity assay

After Nsp1 transfection, HeLa cell were transfected with empty vector or Nsp1 plasmid and 12-well plates (500 cells/well) were seeded and allowed to proliferate for 2 weeks. After treatment, cells were washed with PBS and fixed using methanol and glacial acetic acid (3:1) with subsequent staining with crystal violet solution (0.5% crystal violet solution in 25% methanol) for 15 min at room temperature. After incubation, plates were washed with water. After drying the plates, colonies were scanned and quantified using NIH ImageJ.

### Tumor sphere formation assay

Tumor sphere formation was done essentially as described. Treated GBM-LN-299, 387, and 3691 CSCs were trypsinized and 500 cells/well were plated in a 96-well plate in triplicate. After 12 days, spheroids were counted manually under an inverted microscope.

### Analytical sucrose gradient fractionation

Analytical sucrose gradient fractionations were performed as described with minor modifications (18). In brief, treated cells were resuspended in RNC buffer (20 mM Tris pH 7.5, 140 mM KCl, 1.5 mM MgCl_2_, 0.5 mM dithiothreitol, 200 mg/mL heparin, 1% Triton), and lysed with 26 G insulin syringe. Then, supernatants corresponding to 250 μg of total RNA were treated with 100 U of RNase I (Thermo Fisher, cat#AM2294) at room temperature for 30 min. Then, treated supernatant were manually layered over a 10–50% sucrose gradient and centrifuged at 55,000 rpm for 50 min in a SW41Ti (Beckman) swinging-bucket rotor. Ten 200 μL fractions were successively collected with subsequent proteins precipitation using 30% TCA. Precipitated proteins were washed thrice with chilled acetone followed by solubilized in 1× RIPA buffer. Finally, samples were prepared by boiling with loading gel buffer and analyzed by western blotting.

### Pum labeling of stalled nascent peptides

HeLa cells with or without Nsp1 transfection were treated with HHT (5 µM) for 10 min followed by emetine (100 µM) and Pum (100 µM) treatment for 15 min to label stalled peptides. Cells were subjected to IP with anti-Pum and immunoblotting with cMyc antibody (D84C12).

### Bioinformatic analysis of cancer gene expression

Pan-cancer analysis of whole genomes (ICGC/TCGA): For the analyses of cMyc relation with other genes, we have separated clinical patient data of 2,583 cancer patients in Pan-cancer analysis of whole genomes from ICGC/TCGA and then samples were separated based on cMyc expression. Among these higher cMyc expressed samples, the relationship of cMyc with ABCE1 expression was analyzed by genetic correlation analysis.

### Athymic nude mice xenograft study

The BALB/c pathogen-free athymic nude mice (4 weeks old; bodyweight 20–22 g) were purchased from Jackson Laboratory (USA). Nude mice were housed in barrier facility with sterile temperature-controlled room on a 12 h light:12 h dark schedule and provided with a standard rodent chow diet and water ad libitum. All animal experiments were performed in accordance with the protocols approved by the Administrative Panel on Laboratory Animal Care (APLAC 32974) at Stanford University and comply with all regulations for ethical conduct of animal research.

For engraftment, 1 × 10^6^ HeLa cells were inoculated subcutaneously into the right flank region, and the mice were monitored for tumor development. When tumors attained a size of 100 mm, mice were randomly assigned to five groups (*n* = 5/group). Nsp1 AAV virus were injected intratumorally. Tumor volumes were monitored throughout the experiment on weekly basis and estimated using the formula [(*W*)2 × *L*]/2, where *W* represents the width (shortest tumor diameter), and *L* represents the length (longest tumor diameter). Tumors were dissected and stored in liquid nitrogen or fixed in 4% formaldehyde for further analysis.

### *Drosophila* husbandry

Fly strain crosses were performed according to standard procedure and were raised at indicated temperature. *Drosophila* NB analyses were performed in larvae at 29 °C at 12 h after larval hatching (AHL) to activate the transgene expression and further raised at 29 °C up to 120 h before dissection. To generate the NB MARCM clone, larvae at 24 h AHL were heat shocked for 1 h at 37 °C and further raised at 25 °C for 96–120 h before dissection. *Drosophila* eye tumor studies were performed at 25 °C. Fly lines used in this study are *GMR*-Gal4, *1407*-*GAL4* (#8751), *UAS*-*VCP*-*RI* (#31968), UAS-*Pelo*-*RI* (#34770), *UAS*-*cNot4*-*RI* (#28775), *UAS*-*ABCE1*-*RI* (#31601), UAS-*ZNF598*-*RI* (#61288), *UAS*-*InR* (#8262), *UAS*-*Yki* (#28815), *Ltn1*-EP (#30116), *ERF1*-EP (#17265), *UAS*-*dMyc* (#9674), *UAS*-*Slpr^CA^* (#58826), *UAS*-*Slpr*-*IR* (#32948), and *UAS*-*p38b*-*IR* (#29405) were obtained from Bloomington *Drosophila* Stock Center at Indiana University. *UAS*-*Wts*-*IR* (#106174), *UAS*-*Pelo*-*RI* (#108606), *UAS*-*Clbn*-*RI* (#103351), and *UAS*-*GCN2*-*RI* (#32664) were obtained from Vienna *Drosophila* Resource Center (VDRC). *UAS*-*ZNF598*.*ORF* (#F001909), *UAS*-*Pelo.ORF* (#F003036), and *UAS*-*ABCE1* (#F001097) were purchased from FlyORF *Drosophila* Stock Center. *UAS*-*Notch*-V5 was kindly provided by Dr Mark Fortini, *UAS*-*VCP* by Dr Paul Taylor, *UAS*-*GCN2*-wt and *GCN2*-CA by Dr Pierre Léopold, and UAS-*eIF2α*-S51A by Dr Hyung Don Ryoo. *UAS*-cMyc-WT and *UAS*-cMyc-5P are generated in this study. FL cDNA fragments encoding mouse cMyc-WT and cMyc-5P were PCR amplified from plasmid templates kindly provided by Drs Sonia Coni and Gianluca Canettieri (47) with primers containing Flag tag at the 5′ end and cloned into *pUAST* vector. Transgenic fly generation was done at Bestgene Inc. We used *w*^1118^ as a wild-type control.

### Immunofluorescence of *Drosophila* brain

To perform larval brain staining, briefly, third instar larvae were dissected in Schneider's medium (Invitrogen) and fixed with 4% formaldehyde in PEM buffer (100 mM piperazine-1,4-bis(2-ethanesulfonic acid) at pH 6.9, 1 mM EGTA, 1 mM MgCl_2_) for 23 min at room temperature. Brains were washed three times with 0.1% PBST (1 × PBS + 0.1% Triton X) and were incubated with primary antibody (anti-Prospero 1:200, and anti-Deadpan 1:1,000) overnight at 4 °C. The samples were washed three times with PBST and incubated with the secondary antibodies (1:200 dilution). Samples were imaged on a Leica SP8 confocal microscope, and NB numbers were counted and quantified in NIH Image J.

### RNA-seq analysis

Five Thoraxes from control and Nsp1 expressing flies (7 days old) were dissected and sent to TB-SEQ, Inc. (www.tbseq.com) for RNA-seq. The RNA-seq results were analyzed using the online database (https://maayanlab.cloud/FlyEnrichr/). Volcano plot depicts the most down-regulated genes (blue) and up-regulated (red) in Nsp1 compared with *w*^1118^. Genes were considered significant with adjust *P*–value of <0.05. Nonsignificant genes are shown in black. Dotplot: KEGG pathway analysis and Gene Ontology (GO) analysis (Biological process) of down-regulated and up-regulated pathways in Nsp1 compared with *w*^1118^. The dot size depicts the number of enriched genes in each pathway or GO terms, and the dot color indicates the adjust *P*-value of the enrichment. The differential gene expression results were visualized using ggplot2 (v3.4.0) in R (4.2.2). The KEGG pathway analysis and GO analysis were performed with Enrichr webtool (https://maayanlab.cloud/FlyEnrichr/).

### Light microscopy of fly eyes

Flies of indicated genotypes were collected (3–5 days old) and immediately imaged under Zeiss microscope. Six to eight females were imaged for each genotype, and images were processed by Fiji NIH Image J.

### Analysis of gene expression by real-time quantitative PCR

We used TRIzol (Invitrogen) to extract mRNA from fly heads and iScript cDNA synthesis kit (Bio-Rad) to synthesize cDNA. Real-time quantitative PCR (qRT-PCR) was performed using SYBR Green.

The sequences of qRT-PCR primers we used are as follows:

cMyc forward: GCATGCCCCTCAACGTGAACTTC

cMyc Reverse: CGTTATGCACCAGAGTTTCGAAGC

dMyc forward: GCATGGCCCTTTACCGCTCTGATCCG

dMyc Reverse: CGTCCACTAACCGAGCGCGATTCG

Slpr forward: CTACAAGGGCTTCGATCCGTTG

Slpr Reverse: GTTTGCCAGCAGCTCTTCATCAG

GCN2 forward: TCCAGAAGCAGGCGCAGAAG

GCN2 Reverse: CGTTGGCTTGTCGTGGGTGAG

p38b forward: CGGCCAGGTCTGCAAGGC

p38b Reverse: CCATGTACACTTGCTGGAACTG

Tubulin Forward: CACACCACCCTGGAGCATTC

Tubulin Reverse: CCAATCAGACGGTTCAGGTTG

### Quantification and statistical analysis

Significance of difference between groups has been measured using one- or two-tailed Student's t tests using GraphPad Prism software. Data are reported as mean ± SD. Statistical values, including number of replicates (*n*), can be found in the figure legends. *P < 0.05, **P < 0.01, ***P < 0.0001. For comparing multiple groups, we used one-way ANOVA test followed by Student–Newman–Keuls posttest.

## Supplementary Material

pgae321_Supplementary_Data

## Data Availability

All data are included in the manuscript and/or supporting information.
