# Correction to: Ribosome stalling during *c-myc* translation presents actionable cancer cell vulnerability

**DOI:** 10.1093/pnasnexus/pgae478

**Published:** 2024-11-04

This is a correction to: Tejinder Pal Khaket, Suman Rimal, Xingjun Wang, Sunil Bhurtel, Yen-Chi Wu, Bingwei Lu, Ribosome stalling during *c-myc* translation presents actionable cancer cell vulnerability, *PNAS Nexus*, Volume 3, Issue 8, August 2024, pgae321, https://doi.org/10.1093/pnasnexus/pgae321

In the originally published version of this article, an incorrect version of Figure 5 was published. The authors note that the errors did not impact the interpretation of the Figure.

The updated Figure includes the following changes:

1. In Figure 5A, the right graph was an incorrectly labeled version of the graph in Fig. S5B. It was mistakenly included and has been removed.
2. In Figure 5B, the following labels have been fixed: 100u to 100 um; 1407>N-V5 to 1407>N-V5/Ctrl
3. In Figure 5D and 5E, the 1407>N-V5; Nsp1 label has been adjusted to better reflect the genotypes they are intended to represent.
4. In figure 5E, a statistical analysis was added.

In addition, on page 7, left column, line 7, the following sentence: “The RNAi of Clbn, VCP, and Listerin (Ltn), homologs of key components of the yeast RQC machinery, all attenuated NSC over- proliferation induced by Notch OE (Figs. 5A and S5A–D, G).” has been changed to: “The RNAi of VCP and Listerin (Ltn), homologs of key components of the yeast RQC machinery, both attenuated NSC over- proliferation induced by Notch OE (Figs. 5A and S5A–D, G).”

