# Supplementary material for: Ribosome stalling during c-myc translation presents actionable cancer cell vulnerability
Source: PNAS Nexus. 2024 Aug 13;3(8):pgae321. doi: 10.1093/pnasnexus/pgae321 (PMC11330866; doi:10.1093/pnasnexus/pgae321)
Supplement: pgae321_Supplementary_Data [file pgae321_supplementary_data.pdf]

Supplementary Information for

Ribosome stalling during *c-myc* translation presents actionable cancer cell vulnerability

Tejinder Pal Khaket, Suman Rimal, Xingjun Wang, Sunil Bhurtel, Yen-Chi Wu, and Bingwei Lu*

Email: bingwei@stanford.edu

This PDF file includes:

Figures S1 to S8

Supplementary figure legends

Supplementary Tables 1, 2, 3

Figure S1

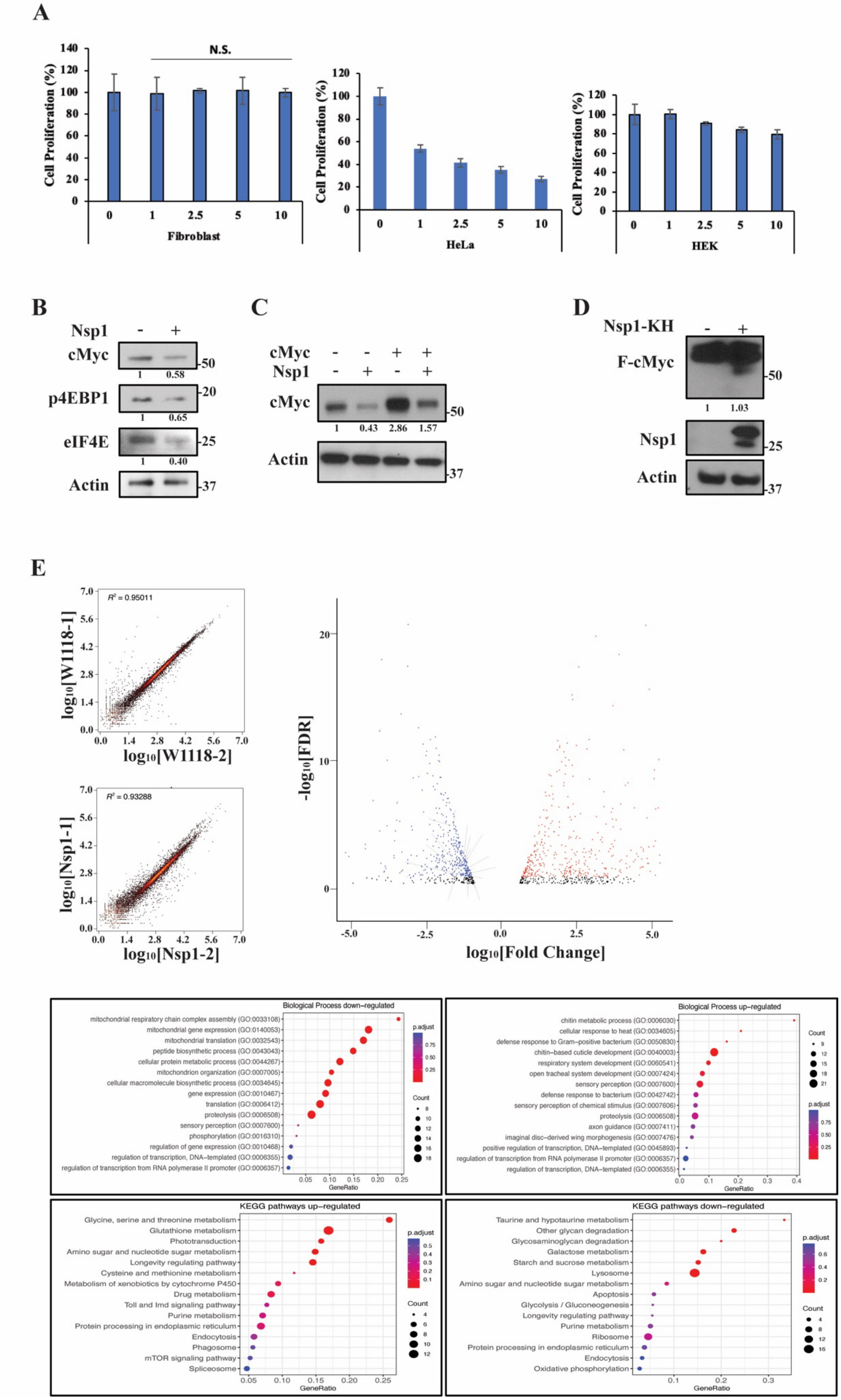
A
N.S.
Cell Proliferation (%)
Fibroblast
HeLa
HEK
0
1
2.5
5
10
B
Nsp1
cMyc
p4EBP1
eIF4E
Actin
1 0.58
1 0.65
1 0.40
C
cMyc
Nsp1
Actin
1 0.43 2.86 1.57
D
Nsp1-KH
F-cMyc
Nsp1
Actin
1 1.03
E
R^2 = 0.95011
R^2 = 0.93288
log10[W1118-1]
log10[W1118-2]
log10[Nsp1-1]
log10[Nsp1-2]
-log10[FDR]
log10[Fold Change]
Biological Process down-regulated
mitochondrial respiratory chain complex assembly (GO:0033108)
mitochondrial gene expression (GO:0140053)
mitochondrial translation (GO:0032543)
peptide biosynthetic process (GO:0043043)
cellular protein metabolic process (GO:0044267)
mitochondrion organization (GO:0007005)
cellular macromolecule biosynthetic process (GO:0034645)
gene expression (GO:0010467)
translation (GO:0006412)
proteolysis (GO:0006508)
sensory perception (GO:0007600)
phosphorylation (GO:0016310)
regulation of gene expression (GO:0010468)
regulation of transcription, DNA-templated (GO:0006355)
regulation of transcription from RNA polymerase II promoter (GO:0006357)
GeneRatio
p.adjust
Count
Biological Process up-regulated
chitin metabolic process (GO:0006030)
cellular response to heat (GO:0034605)
defense response to Gram-positive bacterium (GO:0050830)
chitin-based cuticle development (GO:0040003)
respiratory system development (GO:0060541)
open tracheal system development (GO:0007424)
sensory perception (GO:0007600)
defense response to bacterium (GO:0042742)
sensory perception of chemical stimulus (GO:0007606)
proteolysis (GO:0006508)
axon guidance (GO:0007411)
imaginal disc-derived wing morphogenesis (GO:0007476)
positive regulation of transcription, DNA-templated (GO:0045893)
regulation of transcription from RNA polymerase II promoter (GO:0006357)
regulation of transcription, DNA-templated (GO:0006355)
KEGG pathways up-regulated
Glycine, serine and threonine metabolism
Glutathione metabolism
Phototransduction
Amino sugar and nucleotide sugar metabolism
Longevity regulating pathway
Cysteine and methionine metabolism
Metabolism of xenobiotics by cytochrome P450
Drug metabolism
Toll and Imd signaling pathway
Purine metabolism
Protein processing in endoplasmic reticulum
Endocytosis
Phagosome
mTOR signaling pathway
Spliceosome
KEGG pathways down-regulated
Taurine and hypotaurine metabolism
Other glycan degradation
Glycosaminoglycan degradation
Galactose metabolism
Starch and sucrose metabolism
Lysosome
Amino sugar and nucleotide sugar metabolism
Apoptosis
Glycolysis / Gluconeogenesis
Longevity regulating pathway
Purine metabolism
Ribosome
Protein processing in endoplasmic reticulum
Endocytosis
Oxidative phosphorylation

Fig. S1. Effect of Nsp1 on cMyc expression and function. (**A**) MTT assays showing dose-dependent toxicity of Nsp1 on cancer cells (HeLa) vs non-cancer cells (HEK293T cells and normal human fibroblasts). Cells transfected with different amounts of Nsp1 plasmid DNA (0, 1, 2.5, 5, 10 μg) were analyzed. (**B**) Immunoblot of cMyc, p-4EBP, and eIF4E in lenti-Nsp1 transfected GBM sphere cells. (**C**) Immunoblot of cMyc and Nsp1 in transfected HeLa cells. (**D**) Immunoblot of cMyc in K164A/H165A mutant Nsp1 transfected HeLa cells. (**E**) RNA-seq analysis of differential expression of genes and analysis of different biological processes upregulated and downregulated in *Mhc-Gal4*>APP.C99 flies with or without Nsp1 co-expression. Results were statistically analyzed by GraphPad Prism software (n.s: not significant; *p<0.05, **p<0.01; ***p<0.001 in Student's *t*-test). Values under the blots indicate relative protein levels.

Figure S2

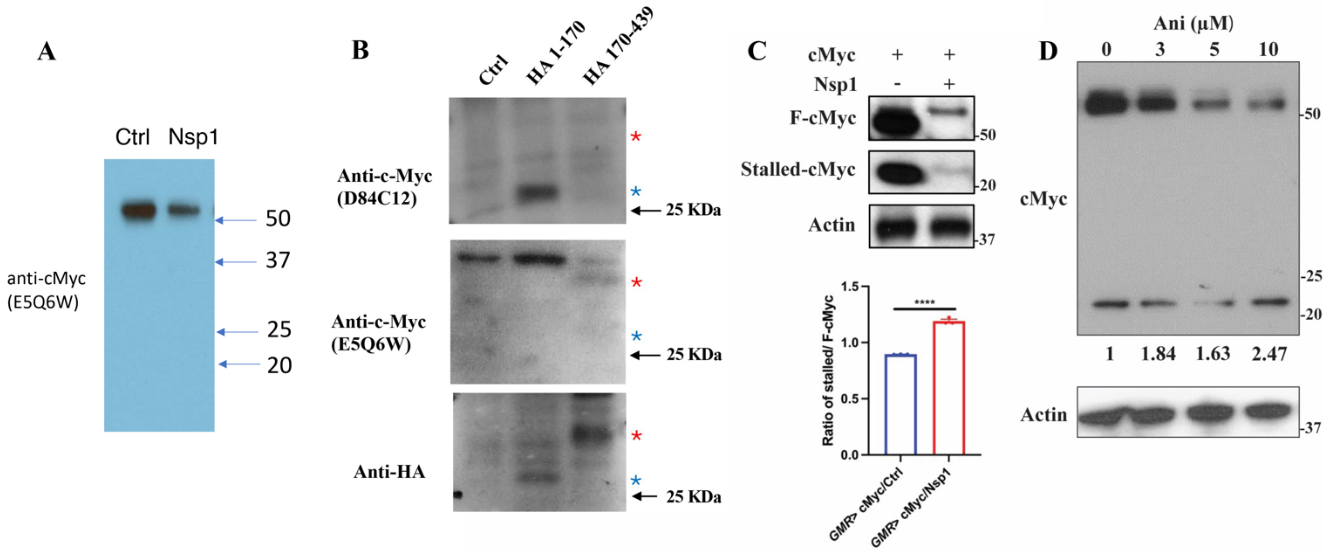

A
Ctrl
Nsp1
50
37
25
20
anti-cMyc
(E5Q6W)
B
Ctrl
HA 1-170
HA 170-439
Anti-c-Myc
(D84C12)
25 KDa
Anti-c-Myc
(E5Q6W)
25 KDa
Anti-HA
25 KDa
C
cMyc + +
Nsp1 - +
F-cMyc
50
Stalled-cMyc
20
Actin
37
Ratio of stalled/ F-cMyc
****
GMR> cMyc/Ctrl
GMR> cMyc/Nsp1
D
Ani (μM)
0 3 5 10
cMyc
-50
-25
-20
1 1.84 1.63 2.47
Actin
-37

Fig. S2. Effect of Nsp1 on cMyc expression under ribosome collision or in tumor condition. (A) Immunoblots showing that the cMyc antibody (E5Q6W) did not recognize the short S-cMyc species in HeLa cell with or without Nsp1 transfection. **(B)** Immunoblots showing specificity of the D84C12 antibody and E5Q6W antibody in recognizing the N-terminal or C-terminal fragment of cMyc. HA-cMyc 1-170 and HA-cMyc 170-439 expressed in HeLa cells were probed with anti-HA antibody and the D84C12 or E5Q6W anti-cMyc antibody. Blue and red asterisks mark the expected position of HA-cMyc 1-170 and HA-cMyc 170-439, respectively. (**C**) Immunoblots and data quantification showing effect of Nsp1 on cMyc level and S-cMyc/FL-cMyc ratio in transgenic fly eyes expressing mammalian cMyc. (**D**) Immunoblots showing concentration-dependent effect of anisomycin induced preferential accumulation of S-cMyc. Values under the blots indicate relative S-cMyc/FL-cMyc ratio. Results were quantified by NIH ImageJ software and statistically analyzed by GraphPad Prism software (***$p<0.001$, ****$p<0.0001$ in Student's *t*-test).

Figure S3

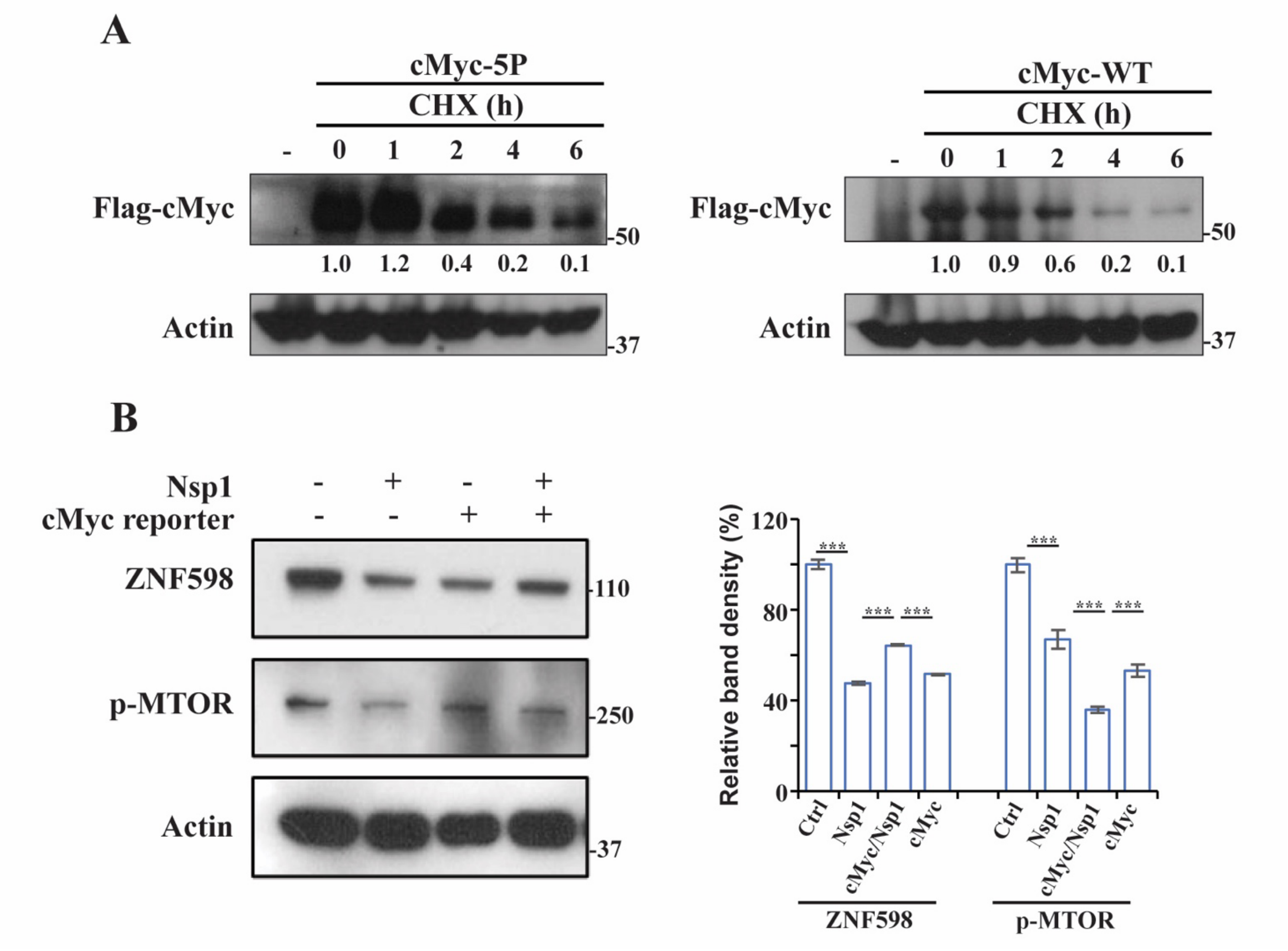
A
cMyc-5P
CHX (h)
- 0 1 2 4 6
Flag-cMyc
-50
1.0 1.2 0.4 0.2 0.1
Actin
-37
cMyc-WT
CHX (h)
- 0 1 2 4 6
Flag-cMyc
-50
1.0 0.9 0.6 0.2 0.1
Actin
-37
B
Nsp1 - + - +
cMyc reporter - - + +
ZNF598
-110
p-MTOR
-250
Actin
-37
Relative band density (%)
120
80
40
0
***
Ctrl
Nsp1
cMyc/Nsp1
cMyc
ZNF598
p-MTOR

Fig. S3. Assessing the effects of 5P mutation on cMyc stability and S-cMyc mimetic on ZNF598 and p-mTOR levels. (**A**) Immunoblots assessing protein stability of cMyc-WT and cMyc-5P in HeLa cells transfected with the corresponding plasmids and treated with cycloheximide to inhibit new protein synthesis in time course experiments measuring protein levels. Values indicate relative protein levels. (**B**) Immunoblots and data quantification showing effect of Nsp1 and stalled cMyc reporter on ZNF598 and p-mTOR expression.

Figure S4

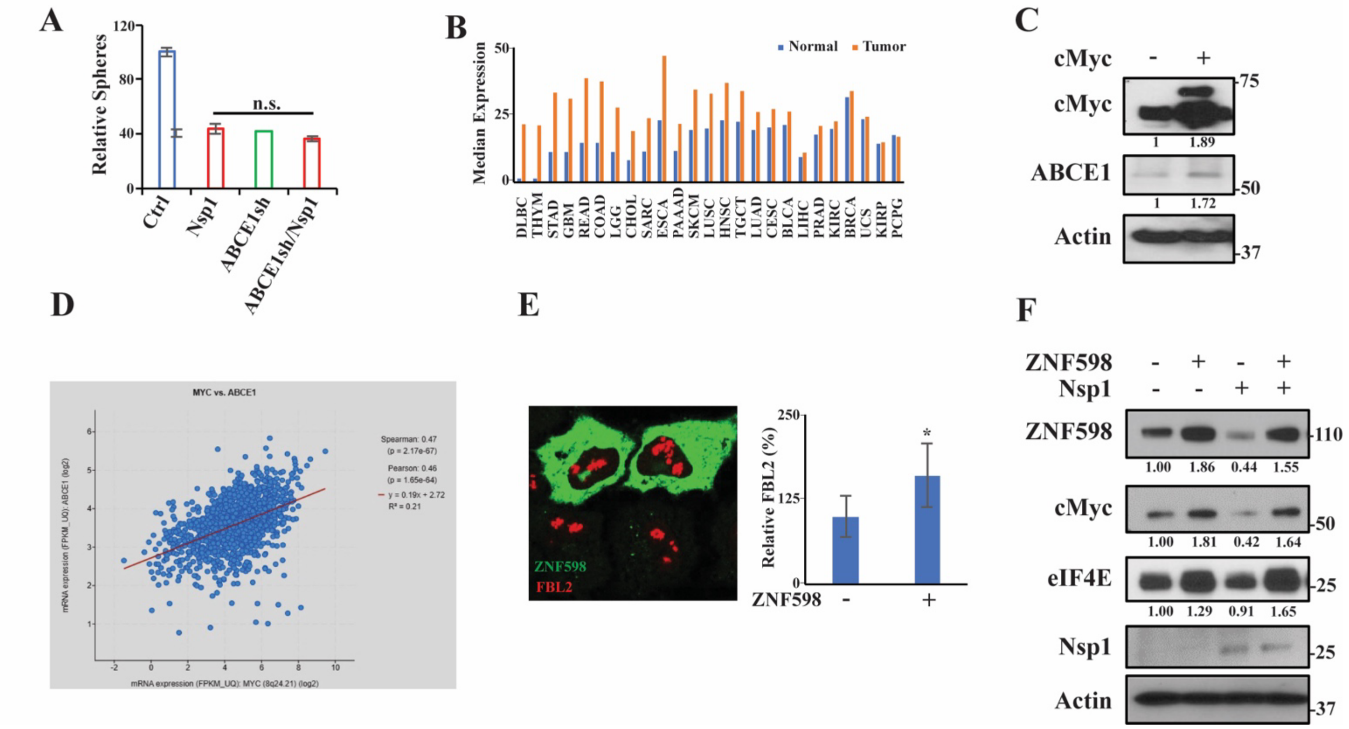
A
Relative Spheres
120
80
40
0
n.s.
Ctrl
Nsp1
ABCE1sh
ABCE1sh/Nsp1
B
Median Expression
50
25
0
Normal
Tumor
DLBC
THYM
STAD
GBM
READ
COAD
LGG
CHOL
SARC
ESCA
PAAAD
SKCM
LUSC
HNSC
TGCT
LUAD
CESC
BLCA
LIHC
PRAD
KIRC
BRCA
UCS
KIRP
PCPG
C
cMyc
-
+
cMyc
75
1
1.89
ABCE1
50
1
1.72
Actin
37
D
MYC vs. ABCE1
E
ZNF598
FBL2
Relative FBL2 (%)
250
125
0
*
ZNF598
-
+
F
ZNF598
Nsp1
ZNF598
110
1.00
1.86
0.44
1.55
cMyc
50
1.00
1.81
0.42
1.64
eIF4E
25
1.00
1.29
0.91
1.65
Nsp1
25
Actin
37

Fig. S4. Functional interaction between Nsp1 and the ZNF598 pathway in cMyc regulation. (A) Effect of ABCE1 RNAi on the anti-growth effect of Nsp1 in GBM neurosphere assay. **(B)** Tumor-wise analysis of ABCE1 expression in normal vs tumor cells based on gene expression profiling and interactive analysis (GEPIA). (**C**) Immunoblots showing effect of cMyc OE on ABCE1 level in HeLa cells. (**D**) Evaluation of the correlation between cMyc and ABCE1 expression among 2583 cancer patients from Pan-cancer analysis of whole genomes (ICGC/TCGA). (**E**) Immunostaining and quantification showing the size of Fibrillarin stained nucleoli in ZNF598 OE HeLa cells. (**F**) Immunoblots showing effect of ZNF598 in attenuating the inhibitory effect of Nsp1 on cMyc and eIF4E protein expression in HeLa cells. Values under the blots indicate relative protein levels.

Figure S5

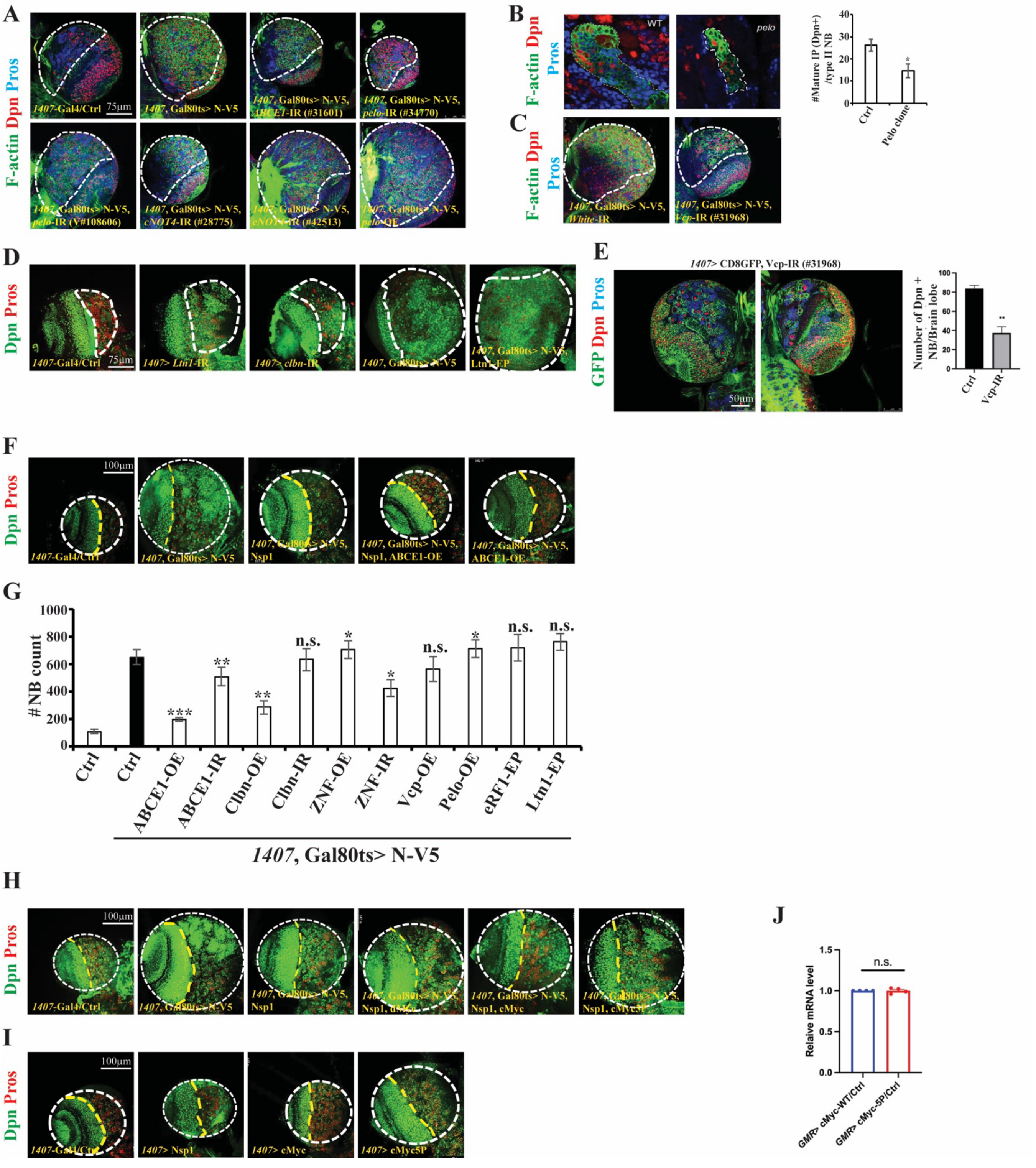

A
F-actin Dpn Pros
1407-Gal4/Ctrl
75μm
1407, Gal80ts> N-V5
1407, Gal80ts> N-V5, ABCE1-IR (#31601)
1407, Gal80ts> N-V5, pelo-IR (#34770)
1407, Gal80ts> N-V5, pelo-IR (V#108606)
1407, Gal80ts> N-V5, cNOT4-IR (#28775)
1407, Gal80ts> N-V5, cNOT4-IR (#42513)
1407, Gal80ts> N-V5, pelo-OE
B
F-actin Dpn Pros
WT
pelo
#Mature IP (Dpn+) /type II NB
Ctrl
Pelo clone
C
F-actin Dpn Pros
1407, Gal80ts> N-V5, White-IR
1407, Gal80ts> N-V5, Vcp-IR (#31968)
D
Dpn Pros
1407-Gal4/Ctrl
75μm
1407> Ltn1-IR
1407> clbn-IR
1407, Gal80ts> N-V5
1407, Gal80ts> N-V5, Ltn1-EP
E
1407> CD8GFP, Vcp-IR (#31968)
GFP Dpn Pros
50μm
Number of Dpn + NB/Brain lobe
Ctrl
Vcp-IR
F
Dpn Pros
100μm
1407-Gal4/Ctrl
1407, Gal80ts> N-V5
1407, Gal80ts> N-V5, Nsp1
1407, Gal80ts> N-V5, Nsp1, ABCE1-OE
1407, Gal80ts> N-V5, ABCE1-OE
G
NB count
n.s.
Ctrl
Ctrl
ABCE1-OE
ABCE1-IR
Clbn-OE
Clbn-IR
ZNF-OE
ZNF-IR
Vcp-OE
Pelo-OE
eRF1-EP
Ltn1-EP
1407, Gal80ts> N-V5
H
Dpn Pros
100μm
1407-Gal4/Ctrl
1407, Gal80ts> N-V5
1407, Gal80ts> N-V5, Nsp1
1407, Gal80ts> N-V5, Nsp1, cMyc
I
Dpn Pros
100μm
1407-Gal4/Ctrl
1407> Nsp1
1407> cMyc
1407> cMyc5P
J
n.s.
Relaive mRNA level
GMR> cMyc-WT/Ctrl
GMR> cMyc-5P/Ctrl

Fig. S5. Nsp1 attenuates tumor proliferation in *Drosophila*. (**A**) Images showing effect of genetic manipulation of RQC factor ABCE1, Pelo, and NOT4 on Notch induced brain tumor phenotype in *Drosophila* larval brain. (**B**) Images showing effect of *pelo* null mutation on brain size and NB number, and MARCM analysis of wild type and *pelo* mutant NB clones showing *pelo* mutation reduced intermediate neuroprogenitor (IP) number without affecting the maintenance of NB. (**C**) Images showing effect of Pelo RNAi on Notch OE induced brain tumor phenotype. (**D**) Images showing effect of genetic manipulation of Listerin (Ltn) and Caliban (Clbn) on NB proliferation in wild type and Notch induced brain tumor conditions. (**E**) Effect of VCP RNAi on NB proliferation in wild type background. (**F**) Images showing effect of Nsp1 and ABCE1 co-expression on NB number in Notch induced brain tumor condition. (**G**) Quantitation of NB counts after genetic manipulation of the various RQC factors in Notch induced brain tumor condition. (**H**) Images showing effect of dMyc, cMyc-WT, and cMyc-5P in attenuating the inhibitory effect of Nsp1 on Notch induced brain tumor phenotype. (**I**) Images showing effect of cMyc, cMyc-5P and Nsp1 expression on brain size and NB number in wild type condition. (**J**) Comparison of transgenic *cMyc-WT* and *cMyc-5P* mRNA levels in the fly eye. ****$p<0.0001$, ***$p<0.001$, **$p<0.01$, *$p<0.05$, n.s., not significant, in Student's *t*-tests or one-way ANOVA test followed by Student–Newman–Keuls post test.

Figure S6

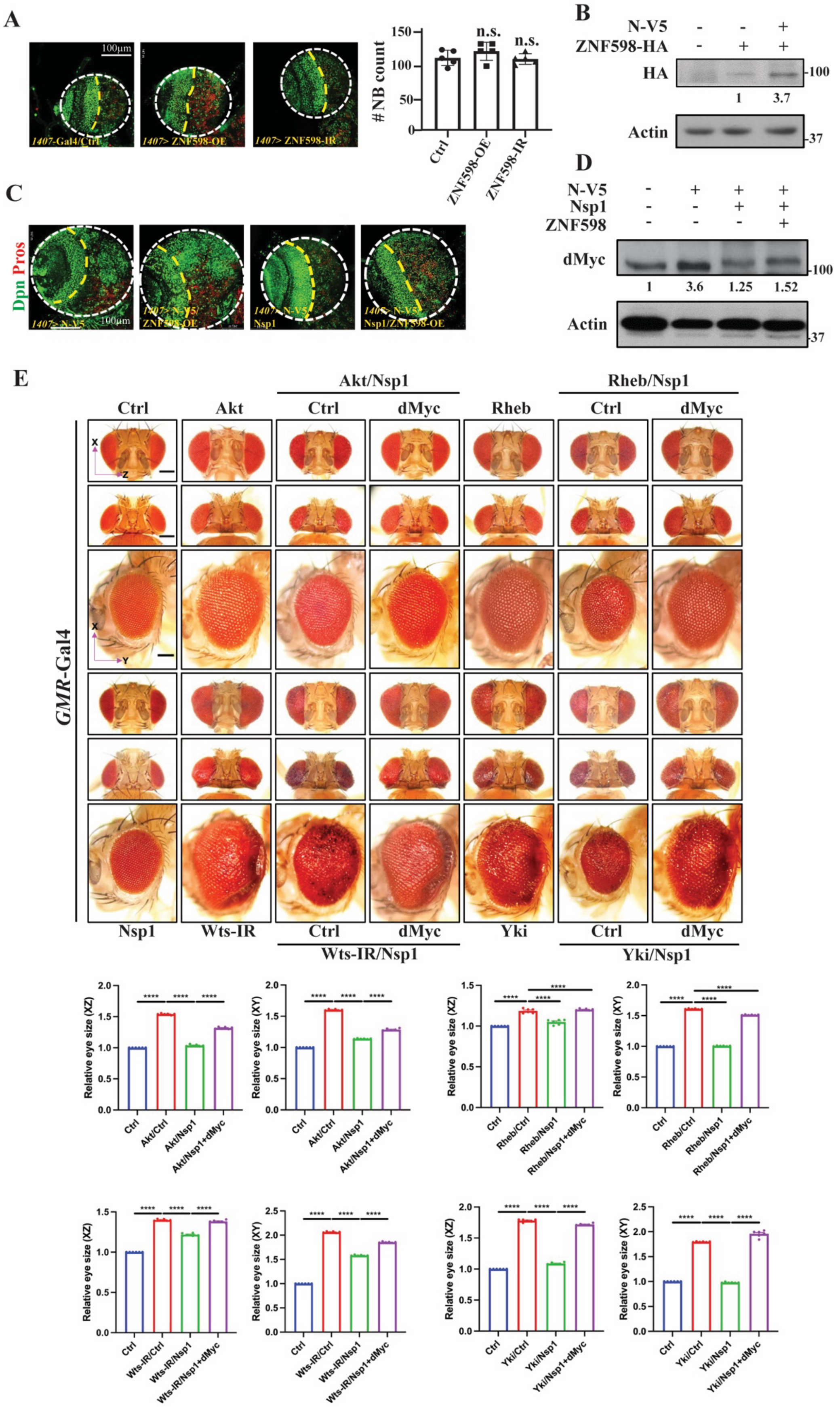
A
100μm
1407-Gal4/Ctrl
1407> ZNF598-OE
1407> ZNF598-IR
NB count
n.s.
n.s.
Ctrl
ZNF598-OE
ZNF598-IR
B
N-V5
ZNF598-HA
HA
Actin
1
3.7
C
Dpn Pros
1407> N-V5
1407> N-V5/ ZNF598-OE
1407> N-V5/ Nsp1
1407> N-V5/ Nsp1/ZNF598-OE
D
N-V5
Nsp1
ZNF598
dMyc
Actin
1
3.6
1.25
1.52
E
Akt/Nsp1
Rheb/Nsp1
Ctrl
Akt
dMyc
Rheb
GMR-Gal4
Nsp1
Wts-IR
Yki
Wts-IR/Nsp1
Yki/Nsp1
Relative eye size (XZ)
Relative eye size (XY)
Akt/Ctrl
Akt/Nsp1
Akt/Nsp1+dMyc
Rheb/Ctrl
Rheb/Nsp1
Rheb/Nsp1+dMyc
Wts-IR/Ctrl
Wts-IR/Nsp1
Wts-IR/Nsp1+dMyc
Yki/Ctrl
Yki/Nsp1
Yki/Nsp1+dMyc

Fig. S6. Effect of the ZNF598 RQC pathway on Myc expression and Myc-driven tumor growth in *Drosophila*. (**A**) Images and data quantification showing effect of genetic manipulation of ZNF598 on brain size and NB count in wild type condition. (**B**) Immunoblots showing effect of Notch OE on the level of HA-tagged ZNF598 expressed from a transgene. (**C**) Images showing effect of ZNF598 OE on Notch induced brain tumor phenotype in the presence or absence of Nsp1 co-expression. (**D**) Immunoblots showing effect of ZNF598 OE on dMyc level in Notch induced brain tumor condition in the presence or absence of Nsp1 co-expression. Values under the blots in **B** and **D** indicate relative protein levels. **(E)** Light microscopy images of the *Drosophila* eye and quantification of eye sizes. Ectopic expression of Akt, Rheb, Wts-IR or Yki transgenes induced eye overgrowth, which was suppressed by the co-expression of Nsp1. Co-expression of dMyc attenuated the anti-growth effect of Nsp1 as indicated by measurement of eye size from XZ and XY dimensions. Genotypes: 1) *GMR-Gal4*/+, 2) *GMR-Gal4*>AKT/+, 3) *GMR-Gal4*>AKT/+; UAS-Nsp1/+, 4) *GMR-Gal4*>AKT/UAS-dMyc; UAS-Nsp1/+, 5) *GMR-Gal4*>Rheb/+, 6) *GMR-Gal4*>Rheb/+; UAS-Nsp1/+, 7) *GMR-Gal4*>Rheb/UAS-dMyc; UAS-Nsp1/+, 8) *GMR-Gal4*/+; UAS-Nsp1/+, 9) *GMR-Gal4*>Wts-IR/+, 10) *GMR-Gal4*>Wts-IR/+; UAS-Nsp1/+, 11) *GMR-Gal4*>Wts-IR/dMyc; UAS-Nsp1/+, 12) *GMR-Gal4*>Yki/+, 13) *GMR-Gal4*>Yki/+; UAS-Nsp1/+, 14) *GMR-Gal4*>Yki/dMyc; UAS-Nsp1/+.

Figure S7

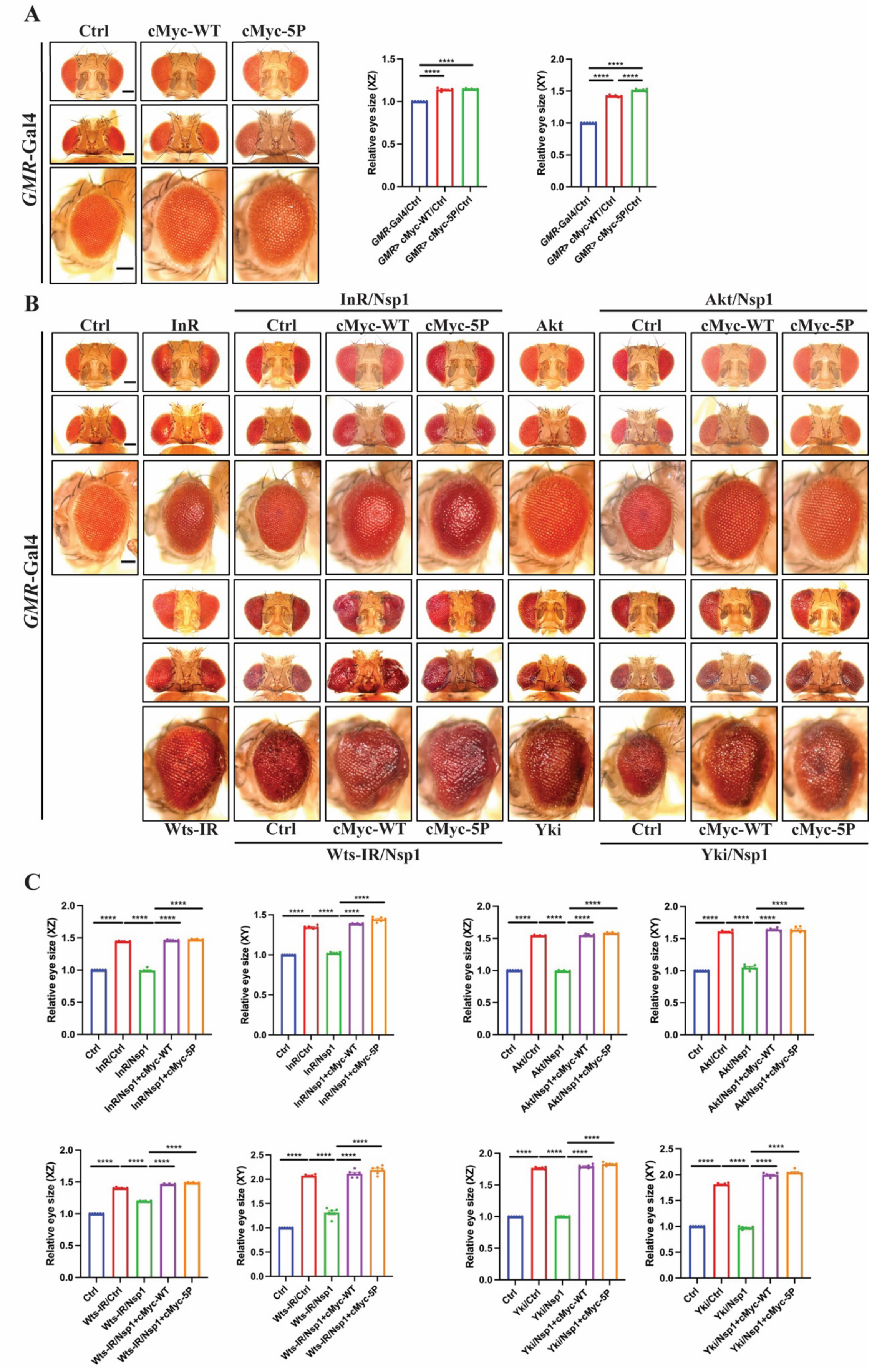
A
Ctrl
cMyc-WT
cMyc-5P
GMR-Gal4
Relative eye size (XZ)
Relative eye size (XY)
GMR-Gal4/Ctrl
GMR> cMyc-WT/Ctrl
GMR> cMyc-5P/Ctrl
B
InR/Nsp1
Akt/Nsp1
Ctrl
InR
cMyc-WT
cMyc-5P
Akt
Wts-IR
Yki
Wts-IR/Nsp1
Yki/Nsp1
C
Ctrl
InR/Ctrl
InR/Nsp1
InR/Nsp1+cMyc-WT
InR/Nsp1+cMyc-5P
Akt/Ctrl
Akt/Nsp1
Akt/Nsp1+cMyc-WT
Akt/Nsp1+cMyc-5P
Wts-IR/Ctrl
Wts-IR/Nsp1
Wts-IR/Nsp1+cMyc-WT
Wts-IR/Nsp1+cMyc-5P
Yki/Ctrl
Yki/Nsp1
Yki/Nsp1+cMyc-WT
Yki/Nsp1+cMyc-5P

Fig. S7. Nsp1 attenuates tumor proliferation in the *Drosophila* eye through Myc. (**A**) Light microscopy images of the fly eye, and eye size quantification showing effect of cMyc or cMyc-5P OE on eye size. The genotypes are: 1) *GMR-Gal4*/+, 2) *GMR-Gal4*>cMyc/+, 3) *GMR-Gal4*>cMyc-5P/+. ****p< 0.0001 in one-way ANOVA test followed by Student-Newman-Keuls post test. Scale bar = 0.4 mm. **(B, C**) Light microscopy images of the *Drosophila* eye (**B**) and quantification of eye sizes (**C**). Expression of InR, Akt, Wts-IR, or Yki induced eye overgrowth, which was suppressed by Nsp1. Co-expression of cMyc-WT or cMyc-5P blocked the anti-growth effect of Nsp1 as indicated by measurement of eye size from XZ and XY dimensions. Genotypes: 1) *GMR-Gal4*/+, 2) *GMR-Gal4*>InR/+, 3) *GMR-Gal4*>InR/+;*UAS*-Nsp1/+, 4) *GMR-Gal4*>InR/*UAS*-cMyc-WT;*UAS*-Nsp1/+, 5) *GMR-Gal4*>InR/*UAS*-cMyc-5P;*UAS*-Nsp1/+, 6) *GMR-Gal4*>Akt/+, 7) *GMR-Gal4*>Akt/+; *UAS*-Nsp1/+, 8) *GMR-Gal4*>Akt/*UAS*-cMyc-WT; *UAS*-Nsp1/+, 9) *GMR-Gal4*>Akt/*UAS*-cMyc-5P; *UAS*-Nsp1/+, 10) *GMR-Gal4*>Wts-IR/+, 11) *GMR-Gal4*>Wts-IR/+; *UAS*-Nsp1/+, 12) *GMR-Gal4*>Wts-IR/*UAS*-cMyc-WT; *UAS*-Nsp1/+, 13) *GMR-Gal4*>Wts-IR/*UAS*-cMyc-5P; *UAS*-Nsp1/+, 14) *GMR-Gal4*>Yki/+, 15) *GMR-Gal4*>Yki/+;*UAS*-Nsp1/+, 16) *GMR-Gal4*>Yki/*UAS*-cMyc-WT; *UAS*-Nsp1/+, 17) *GMR-Gal4*>Yki/*UAS*-cMyc-5P; *UAS*-Nsp1/+. ****p< 0.0001 in one-way ANOVA test followed by Student-Newman-Keuls post test. Scale bar = 0.4 mm.

Figure S8

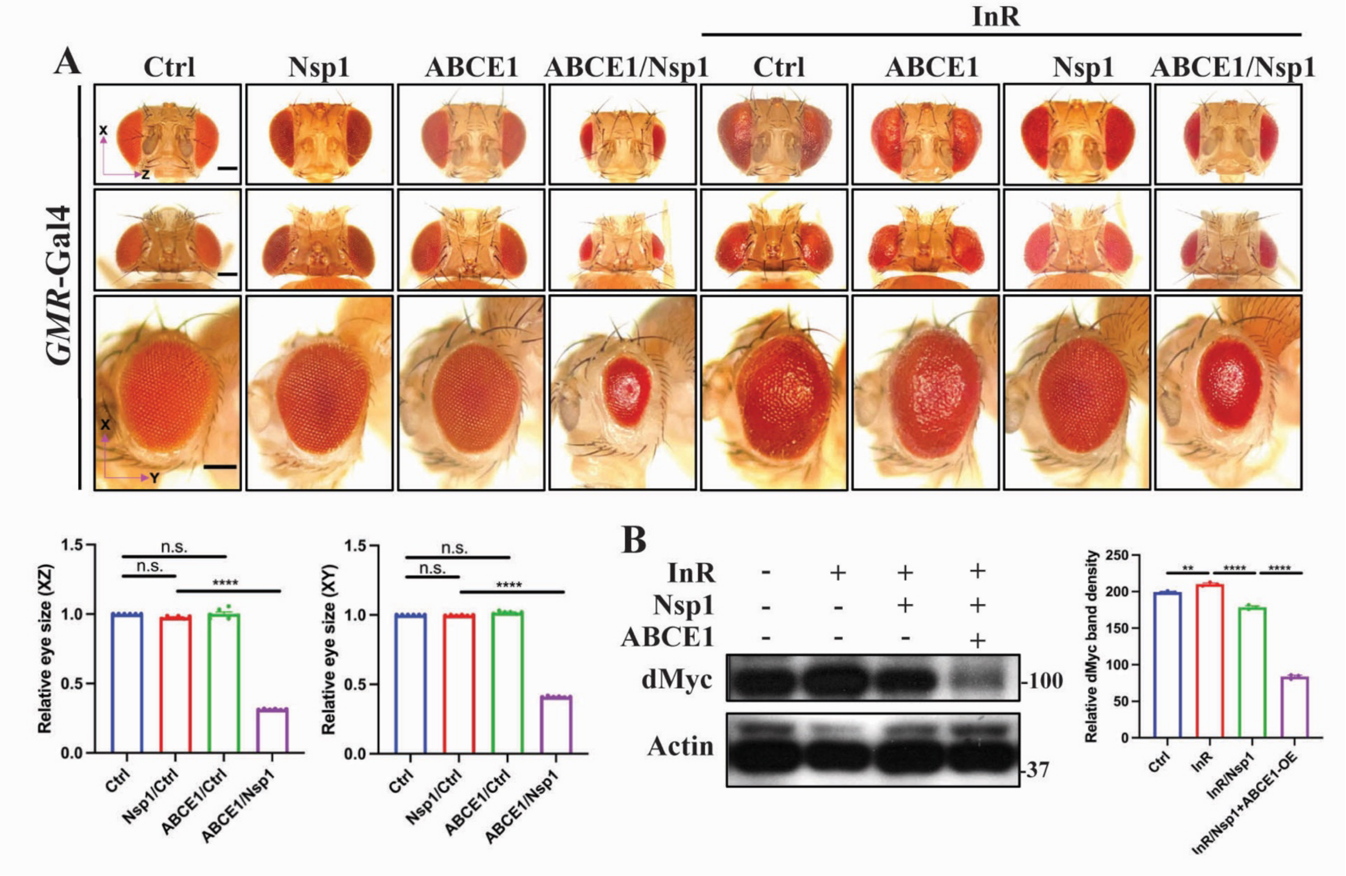
A
InR
Ctrl
Nsp1
ABCE1
ABCE1/Nsp1
Ctrl
ABCE1
Nsp1
ABCE1/Nsp1
GMR-Gal4
Relative eye size (XZ)
Relative eye size (XY)
n.s.
****
Ctrl
Nsp1/Ctrl
ABCE1/Ctrl
ABCE1/Nsp1
B
InR
Nsp1
ABCE1
dMyc
Actin
-100
-37
Relative dMyc band density
Ctrl
InR
InR/Nsp1
InR/Nsp1+ABCE1-OE

Fig. S8. **Nsp1 attenuates tumor proliferation in the fly eye through ABCE1 and dMyc.** (**A**) Light microscopy images of the fly eye and quantification of eye sizes. Expression of Nsp1 and ABCE1 alone did not produce obvious eye phenotype. When co-expressed, Nsp1 and ABCE1 induced a small eye phenotype in a wild type background or InR OE condition. Eye size was quantified by measurement from XZ and XY dimensions. Genotypes: 1) *GMR-Gal4*/+, 2) *GMR-Gal4*/+; UAS-Nsp1/+, 3) *GMR-Gal4*/+; UAS-ABCE1/+, 4) *GMR-Gal4*/+; UAS-Nsp1/UAS-ABCE1, 5) *GMR-Gal4*>InR/+, 6) *GMR-Gal4*>InR/+; UAS-ABCE1/+, 7) *GMR-Gal4*>InR/+; UAS-Nsp1/+, 8) *GMR-Gal4*>InR/+; UAS-Nsp1/UAS-ABCE1. (**B**) Immunoblot analysis of *Drosophila* heads and data quantification showing that expression of Nsp1 attenuated InR induced dMyc upregulation. Co-expression of ABCE1 further reduced dMyc protein level. n.s: not significant, ** $p<0.01$, ****$p< 0.0001$ in one-way ANOVA test followed by Student-Newman-Keuls post test. Scale bar = 0.4 mm.

Supplementary Table 1: List of Antibodies

**Antibodies**	**Product ID**	**Manufacturer**
Nsp1 SARS-CoV2	DA103	MRC PPU
Human Phospho-GCN2 (T899) Antibody	AF7605-SP	RND systems
Phospho-eIF2S1 (Ser51) Polyclonal antibody	28740-1-AP	Proteintech
Phospho-mTOR (Ser2448) Monoclonal antibody	67778-1-Ig	Proteintech
Red fluorescent protein Monoclonal antibody	67378-1-Ig	Proteintech
GFP tag Monoclonal antibody	66002-1-Ig	Proteintech
ZAKa Polyclonal antibody	14945-1-AP	Proteintech
Anti-ZNF598 antibody produced in rabbit	HPA041760	Sigma
cMyc (D84C12) Rabbit mAb #5605	5605S	CST
FBL Polyclonal antibody	16021-1-AP	Proteintech
RPS10 Polyclonal antibody	14894-1-AP	Proteintech
MYC tag Monoclonal antibody mouse	60003-2-Ig	Proteintech
Phospho-Akt (Thr308) (244F9) Rabbit mAb #4056	4056S	CST
Anti-ZNF598 antibody produced in rabbit	HPA041760	Sigma
DYKDDDDK tag monoclonal antibody (Binds to FLAG® tag epitope)	66008-4-Ig	Proteintech
ABCE1 Polyclonal antibody	28548-1-AP	Proteintech
Anti-phospho-H2A.X (Ser139) Antibody	07-164-25	Sigma
HA Tag Monoclonal antibody	66006-2-Ig	Proteintech
cMyc (E5Q6W) Rabbit mAb	# 18583	CST
eIF4E monoclonal antibody	66655-1-Ig	Proteintech
Phospho-AKT (Ser473) monoclonal antibody	66444-1-Ig	Proteintech
RACK1; GNB2L1 monoclonal antibody	66940-1-Ig	Proteintech
Phospho-Akt (Thr308) (244F9) Rabbit mAb #4056	4056S	CST
Phospho-mTOR (Ser2448) monoclonal antibody	67778-1-Ig	Proteintech
Phospho-Akt (Thr308)	4056S	CST
Rabbit anti-Deadpan		Jan Lab/UCSF
Mouse anti-Prospero	Prospero(MR1A)	DSHB
β-actin	66009-1-Ig	Proteintech
Anti-Puromycin,	MABE343	Millipore
Alexa Fluor™ 488	A11001	Invitrogen
Alexa Fluor™ 546	A11035	Invitrogen
Alexa Fluor™ 633	21052	Invitrogen

Supplementary Table 2: List of shRNA, siRNA, and overexpression plasmids

Vectors	Product ID	Manufacturer
pCDNA4 CoV-2 Nsp1 3xFLAG	176057	Addgene
pCDNA4TO K164A H165A CoV-2 Nsp1 3xFLAG	176060	Addgene
pLVX-EF1alpha-SARS-CoV-2-Nsp1-2xStrep-IRES-Puro	141367	Addgene
Lenti-sh1368 knockdown c-myc	29435	Addgene
Znf598siRNA	AM16708	Invitrogen
ZNF598 Lentiviral Vector (Human) (CMV) (pLenti-GIII-CMV)	51654061	Abm
PDK1 siRNA/shRNA/RNAi Lentivector (Human) (Target b)	363200910595	Abm
Myc Lentiviral Vector (Human) (EF1a) (pLenti-GIII-EF1a)	31206062	Abm
EIF2AK4 (GCN2) siRNA	SASI_Hs01_00097889	Sigma
pcDNA4/TO/GFP-ZNF598	141191	Addgene
pcDNA4/TO/Strep-HA-ZAK alpha	141193	Addgene
pCDH-puro-myr-HA-Akt1	46969	Addgene
pHRIG-Akt1	53583	Addgene
GFP-P2A-MYC-P2A-RFP	SC1017	GenScript
GFP-P2A-MYC5P-P2A-RFP	SC1017	GenScript
ABCE shRNA	110670910395	Abm
pCDNA3-HA-HA-human CMYC (1-170)	#203426	Addgene
pCDNA3-HA-HA-human CMYC (1-208)	#203427	Addgene
pCDNA3-HA-HA-human CMYC (1-277)	#203428	Addgene
pCDNA3-HA-HA-human CMYC (170-439)	#203430	Addgene
pCDNA3-HA-HA-human CMYC (208-439)	#203433	Addgene
pCDNA3-HA-HA-human CMYC (277-439)	#203435)	Addgene

Supplementary Table 3. Chemicals

Chemicals	Product ID	Source
NuPAGE™ 4 to 12%, Bis-Tris, 1.0 mm, Mini Protein Gel, 10-well	NP0321BOX	Invitrogen
NuPAGE™ MOPS SDS Running Buffer (20X)	NP0001	ThermoFisher
Western Blot Stripping Buffer	T7135A	Takara Bio Inc.
4x Laemmli Sample Buffer	1610747	BioRad
Lipofectamine™ 2000 Transfection Reagent	11668027	ThermoFisher
Opti-MEM™ I Reduced Serum Medium	31985070	ThermoScientific
PS210	HY-121629	MCE
PS48	ab142133	ABCAM
MK-2206 2HCl	HY-10358	Medchemexpress
SC79	HY-18749	Medchemexpress
Polybrene	TR-1003-G	Sigma
QIAGEN Plasmid Maxi Kit (10)	12162	Qiagen
Protease Inhibitor Cocktail	P8340-1ML	Sigma
Lenti-X™ Concentrator	631231	Takara
NP-40 Surfact-Amps™ Detergent Solution	85124	ThermoFisher
ProLong™ Gold Antifade Mountant	P10144	ThermoFisher
DMEM/F-12, no glutamine	21331020	ThermoFisher
GlutaMAX™ Supplement	35050061	ThermoFischer
B-27™ Supplement (50X), minus vitamin A	12587010	ThermoFisher
Anisomycin	S7409	SELLECK Chemicals
ISRIB	SML0843	SIGMA
GCN2-IN-1	36027	Cayman
SB203580	19-135	SIGMA
iScript cDNA Synthesis kit	1708890	Biorad
Protein A/G Plus -Agarose	Sc-2003	Santa Cruz
RIPA Lysis Buffer	20-188	Millipore
Phosphatase Inhibitor Cocktail	K1015	ApexBT
Sodium Bicarbonate 7.5% solution	25080094	ThermoFisher
Sodium Pyruvate (100 mM)	11360070	ThermoFisher
DH5α Competent Cells	18265017	ThermoFischer
DAPI	5748	Tocris
Invitrogen™ UltraPure™ DNase/RNase-Free Distilled Water	10-977-015	ThermoFischer
N-2 Supplement (100X)	17502048	ThermoFisher

Human FGF-basic 154 aa (FGF2)	100-146AF	Shenandoah Biotechnology
RNase I	AM2294	ThermoFisher
